# Is Empathy for Pain Unique in Its Neural Correlates? A Meta-Analysis of Neuroimaging Studies of Empathy

**DOI:** 10.3389/fnbeh.2018.00289

**Published:** 2018-11-27

**Authors:** Inge Timmers, Anna L. Park, Molly D. Fischer, Corey A. Kronman, Lauren C. Heathcote, J. Maya Hernandez, Laura E. Simons

**Affiliations:** Department of Anesthesiology, Perioperative, and Pain Medicine, Stanford University School of Medicine, Palo Alto, CA, United States

**Keywords:** empathy, empathy for pain, ALE meta-analysis, functional imaging, brain imaging

## Abstract

Empathy is an essential component of our social lives, allowing us to understand and share other people's affective and sensory states, including pain. Evidence suggests a core neural network—including anterior insula (AI) and mid-cingulate cortex (MCC)—is involved in empathy for pain. However, a similar network is associated to empathy for non-pain affective states, raising the question whether empathy for pain is unique in its neural correlates. Furthermore, it is yet unclear whether neural correlates converge across different stimuli and paradigms that evoke pain-empathy. We performed a coordinate-based activation likelihood estimation (ALE) meta-analysis to identify neural correlates of empathy, assess commonalities and differences between empathy for pain and for non-pain negative affective states, and differences between pain-empathy evoking stimuli (i.e., facial pain expressions vs. acute pain inflictions) and paradigms (i.e., perceptual/affective vs. cognitive/evaluative paradigms). Following a systematic search, data from 128 functional brain imaging studies presenting whole-brain results of an empathy condition vs. baseline/neutral condition were extracted. Synthesizing neural correlates of empathy confirmed a core network comprising AI, MCC, postcentral gyrus, inferior parietal lobe, thalamus, amygdala, and brainstem. There was considerable overlap in networks for empathy for pain and empathy for non-pain negative affective states. Important differences also arose: empathy for pain uniquely activated bilateral mid-insula and more extensive MCC. Regarding stimuli, painful faces and acute pain inflictions both evoked the core empathy regions, although acute pain inflictions activated additional regions including medial frontal and parietal cortex. Regarding paradigms, both perceptual/affective and cognitive/evaluative paradigms recruited similar neural circuitry, although cognitive/evaluative paradigms activated more left MCC regions while perceptual/affective paradigms activated more right AI. Taken together, our findings reveal that empathy for pain and empathy for non-pain negative affective states share considerable neural correlates, particularly in core empathy regions AI and MCC. Beyond these regions, important differences emerged, limiting generalizability of findings across different affective/sensory states. Within pain-empathy studies, the core regions were recruited robustly irrespective of stimuli or instructions, allowing one to tailor designs according to specific needs to some extent, while ensuring activation of core regions.

## Introduction

Empathy is an essential part of being a social organism. It allows one to understand and share emotions and promotes prosocial behavior (Davis, 1994; Preston and De Waal, 2002; Singer and Lamm, 2009; Zaki, 2014; Decety et al., 2016). For instance, when a parent sees his/her child feeling sad or in pain, empathy allows them to understand what the child is experiencing, share that feeling, and respond in an appropriate manner (e.g., with comfort or encouragement). Although there are different views on the core components of empathy, there is some agreement that it involves (1) awareness and understanding of the other person's emotions. This part is similar to mentalizing and theory of mind, where one is explicitly making sense of another person's affective state or beliefs/intentions (Leslie et al., 2004); (2) distinction between the other person and the self; and (3) sharing of the other person's affective state (similar, but distinct from emotional contagion, which refers to the catching and automatic mimicking of other people's emotions; Hatfield et al., 1993). These components together refer to the “isomorphic” representation of the other person's affective state. Some argue that empathy also comprises feelings of compassion or sympathy (also referred to as empathic care/concern) and the prosocial motivation to help the other person (see Singer and Lamm, 2009; Zaki and Ochsner, 2012, for more discussion).

An extensive body of literature is available on the neural correlates of empathy. Several reviews and systematic analyses have synthesized data and identified a core network for empathy that includes the anterior insula (AI) and the anterior mid-cingulate cortex (aMCC; sometimes referred to as the dorsal anterior cingulate cortex, dACC) (Fan et al., 2011; Lamm et al., 2011; Bzdok et al., 2012). These regions work in synchrony with other brain regions that are associated with related sub-concepts, such as the medial prefrontal cortex (mentalizing) and temporoparietal junction (self-other distinction) (see Lamm et al., 2017). There is also considerable overlap between the regions activated during the experience of certain affective experiences and those activated during the observation of someone else experiencing them (see e.g., Baird et al., 2011; Lamm et al., 2011; Zaki et al., 2016). This mirror-like concept has been described for other mental processes as well, and is most well-known in the realm of sensory-motor systems where it enables us and other animals to understand others' actions (Rizzolatti and Craighero, 2004).

Whether empathy for pain should be considered a separate entity is unclear. Some studies and meta-analyses on the neural correlates of empathy do not include empathy for pain, arguing that pain is not a classic emotion (e.g., Bzdok et al., 2012). Other studies, however, use a mixture of emotions such as anger, sadness, and pain to elicit empathy, without explicitly separating results (Brunnlieb et al., 2013). In any case, it is clear that observing—or even merely anticipating—someone else in pain elicits an empathic response (Decety and Lamm, 2006; Bernhardt and Singer, 2012; Lamm et al., 2017). A meta-analysis also found that neural activation for observing someone else in pain overlaps with the direct experience of pain (Lamm et al., 2011), including recruitment of AI, aMCC, and precuneus. This core network is in line with what has been found for empathy when pain is excluded (Bzdok et al., 2012), and when both pain and non-pain studies are included (Fan et al., 2011). Studies that have compared pain with other affective stimuli have also found commonalities. For instance, Benuzzi et al. (2008) found that several regions responded to both the observation of painful and disgusting situations, including the mid-, posterior and perigenual cingulate cortex, the insula, parietal operculum, and superior frontal gyrus. The authors suggested that the overlap may underlie affective and motor reactions to aversive stimuli. Simon et al. (2006) also identified overlap in neural activation patterns to pain and angry stimuli, including activation in the amygdala, superior temporal sulcus (STS), and ACC. However, when the authors contrasted empathy for pain and empathy for anger directly, they observed several differences, including in the AI, where pain faces evoked an increased neural response compared to angry faces. Taken together, this begs the question whether empathy for pain and empathy for other non-pain affective states rely on the same underlying neural circuitry. This information is especially relevant when investigating empathy in a specific context or state, such as chronic pain, acute procedural pain in health care, depression, anxiety disorders, and it is important to know whether neural correlates of empathy for the specific state are unique or similar to empathy for other (potentially co-morbid) affective or sensory states. Methodologically, having a participant observing someone else in pain is a commonly used and powerful way to investigate correlates of empathy in an experimental context, but it is unclear whether findings from empathy for pain studies generalize to empathy for other affective states, and vice versa. A meta-analytic comparison of empathy for pain and empathy for non-pain negative affective states is therefore a major aim of the current study.

Given the vast number of sensory and affective states that can be observed, there are many ways to elicit an empathic response in an experimental setting. Unsurprisingly, not all empathy paradigms yield the same results. For instance, it has been found that observing pictures of an acute pain infliction activates a different neural network than a more abstract cue-based (real-life) pain empathy paradigm involving inferring and representing mental states of the other person (Lamm et al., 2011). Another common way to elicit an empathic response is to present the participant with photos or videos of facial or bodily expressions, which may be particularly able to capture a full empathic response when context is relevant (e.g., when investigating empathy for chronic instead of empathy for acute pain or when studying empathy for a loved one). One study employed both pain facial expressions and pain infliction to body parts (Danziger et al., 2009). Although the authors did not perform a direct comparison, their results point toward a more widespread network for the pain infliction paradigm, while the faces elicited demarcated activity in MCC and AI that was furthermore more left-lateralized. In addition to using different stimuli, studies have been using different paradigms and instructions. For instance, some studies report on empathic responses that are evoked automatically when observing another individual in a specific sensory or affective state, without any specific instructions (e.g., passive viewing or using a distraction task). Other studies do use specific instructions to, for instance, evaluate another person's feelings or the level of pain the other person is experiencing. A recent meta-analysis (Fan et al., 2011) contrasted these types of paradigms, referred to perceptual/affective when there are no explicit instructions on empathy vs. cognitive/evaluative when there are explicit instructions to perform an empathy task. The authors revealed a similar left lateralization, as the perceptual/affective paradigms elicited bilateral AI, while cognitive/evaluative paradigms elicited left AI and more extensive activation in MCC (Fan et al., 2011). In the current study, we aim to replicate these findings and extend them to empathy for pain specifically, performing a systematic comparison between cognitive/evaluative and perceptual/affective paradigms in empathy (for pain and non-pain), and in empathy for pain specifically. In addition, we aim to examine the use of facial expressions as stimuli to elicit empathy for pain in comparison to using acute pain infliction paradigms.

In summary, the aim of the current study is three-fold: (1) to replicate previous findings identifying a core neural network for empathy, (2) to understand shared and unique neural representations of empathy for pain in comparison to empathy for non-pain negative affective states, and (3) to investigate whether neural correlates are similar when studies use different stimuli (i.e., facial pain expressions vs. acute pain inflictions) and different paradigms (i.e., perceptual/affective vs. cognitive/evaluative paradigms). Accordingly, we perform a meta-analysis of neuroimaging studies of empathy using a coordinate-based activation likelihood estimation (ALE) analysis.

## Methods

### Study selection

Relevant studies were identified through searches in PubMed, Embase, Cochrane Library, and PsychInfo using the keywords [“empathy” OR “empath^*^”] AND [“MRI” OR “imaging” OR “PET”], plus corresponding variations and mesh terms (see Supplementary Material for full search terms developed in partnership with Stanford Lane Medical Library). The search was performed in December 2017. Studies were selected if they were written in the English language and involved human participants. There were no criteria for year of publication. Reference lists of relevant articles, reviews and meta-analyses were inspected to identify any further relevant studies. Screening was performed by two independent people per article (IT, AP, MF, CK), and any disagreements were discussed to reach consensus.

Studies were selected for a full text review if they reported on healthy participants (or a healthy control group), presented original research, and used functional imaging (fMRI or PET) during an empathy task (see Table 1). Similar to Fan et al. (2011), we defined a task as an empathy task if it required participants to observe the emotional or sensory state of others (with or without an explicit instruction to do so), to share the emotional or sensory state of another person and make a subsequent judgement or evaluation, or to imagine what another person is feeling. Additional inclusion criteria were that the paper performed a whole brain analysis (instead of region of interest analyses only) and reported coordinates of contrasts between an empathic condition vs. baseline or a neutral condition. Studies that included pharmacological or psychological interventions and did not report on placebo or pre-intervention data were not included. Full text review was performed independently by two members of the research team (IT, CK) and any disagreements were discussed to reach consensus. Data extraction was performed by AP and MF, and reviewed by IT and CK.

**Table 1:** Systematic review components in PICOS format (population, intervention, comparators, outcomes, and study designs).

**Population**	Healthy participants (no psychiatric or neurologic conditions), all age ranges. (nb. Participants may be from a healthy control group or a placebo group in a study including a separate clinical group).
**Intervention**	All studies using **an empathy task** An empathy task is defined as one in which the instructions required participants: - to observe the emotional or sensory state of others (with or without an explicit instruction to do so), - to share the emotional or sensory state of another person and make a subsequent judgement or evaluation, or - to imagine what another person is feeling
**Comparators**	Neural correlates of: - empathy in general - empathy for pain vs. empathy for non-pain negative affective states - empathy for pain using different stimuli (facial pain expressions vs. acute pain inflictions) - empathy using different paradigms (cognitive/evaluative vs. affective/perceptual empathy) - empathy for pain using different paradigms (cognitive/evaluative vs. affective/perceptual empathy)
**Outcomes**	Neural activation foci (coordinates of relevant contrasts in MNI space) and sample size
**Study Designs**	Original research (reviews or abstracts not included) Functional imaging (fMRI or PET) Whole brain analysis of activation patterns (region of interest analysis only or functional connectivity analyses not included) Coordinates presented between empathy condition and baseline or neutral condition (group analyses, correlation analyses, comparison of two empathy conditions not included^*^) No manipulation that may bias results (e.g., pharmacological challenge or priming)

*MNI, Montreal Neurologic Institute*;

* *As our definition of an empathy task included tasks without explicit instructions (i.e., passive watching/distraction tasks), paradigms using high-order baselines were excluded (e.g., making a gender judgement of facial pain expressions)*.

### Data analysis

The following data was extracted from all included studies: the MNI coordinates of all relevant contrasts that were reported as significant by the corresponding studies (Talairach coordinates were converted into MNI coordinates) and sample size. The studies were further characterized based on their experimental protocol: empathy for pain vs. empathy for non-pain affective states^1^; perceptual/affective vs. cognitive/evaluative empathy; facial painful expression or acute pain infliction stimuli (pain studies only).

Extracted foci were inputted to GingerALE 2.6.3 (http://www.brainmap.org/ale/) to calculate the activation likelihood estimations (ALE) for each voxel in the brain (Eickhoff et al., 2009, 2012; Turkeltaub et al., 2012). GingerALE uses a random-effects algorithm to identify which brain areas show convergence of activation foci across different studies, taking into account the sample sizes of each study. In addition to examining convergence across all empathy-related studies, we performed several contrasts to test for statistical differences across convergence (Eickhoff et al., 2011). The following contrasts were performed: (a) empathy for pain vs. empathy for non-pain negative affective states (i.e., excluding empathy for positive affective states); (b) facial pain expressions vs. acute pain infliction to a body part (pain studies only); (c) cognitive/evaluative empathy vs. affective/perceptual empathy (all studies), and (d) cognitive/evaluative empathy vs. affective/perceptual empathy (pain studies only). Output images were thresholded to a corrected *p* < 0.05 level using a cluster-level inference [5,000 permutations, initial cluster-forming threshold of FDR *p* < 0.01]. The Talairach Daemon was used to extract anatomical labels for the identified clusters (MNI coordinates were transformed to Talairach), which were individually verified.

## Results

### Study selection and characteristics

**Figure 1:**
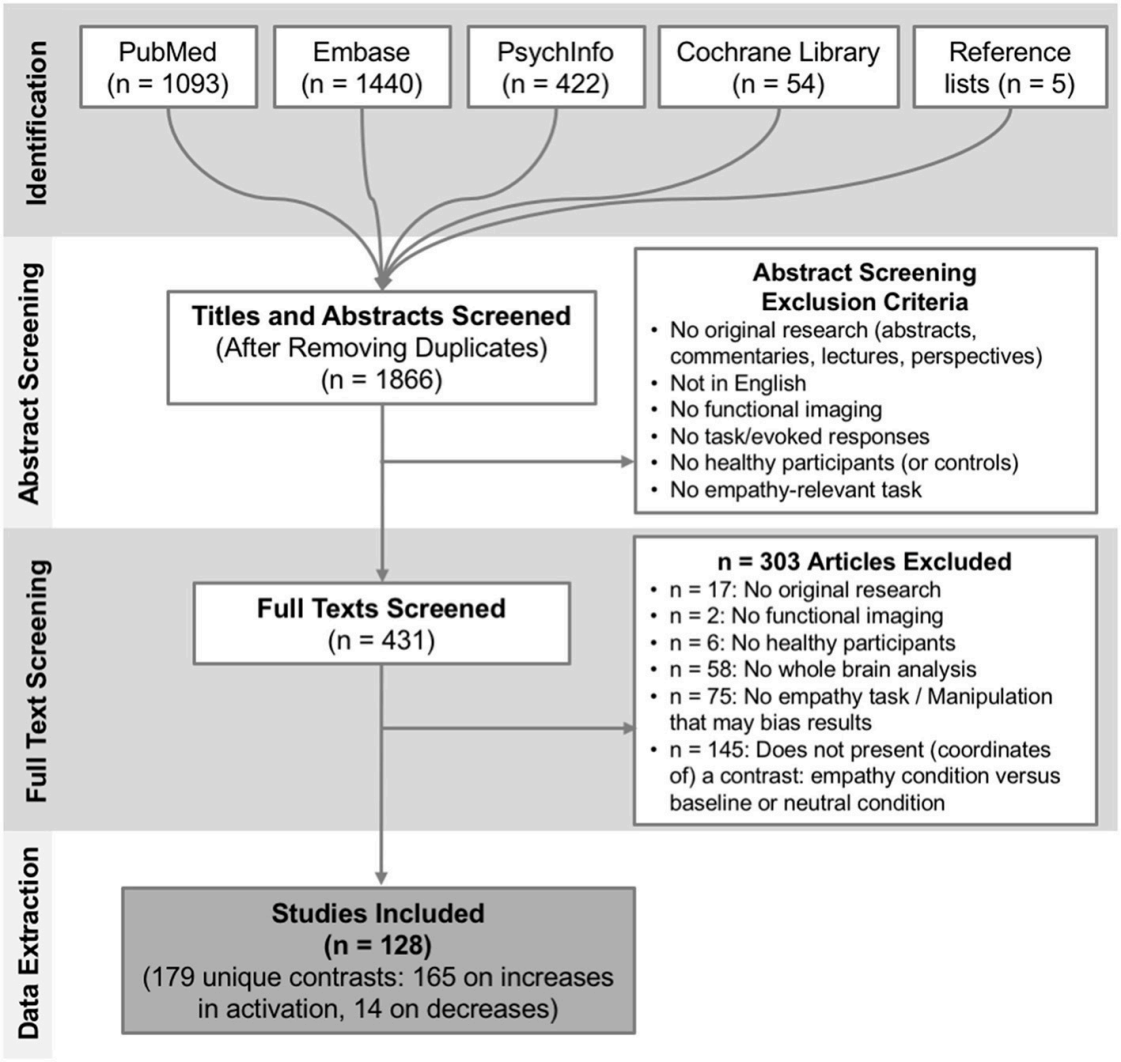
Overview of the study selection process. Depicted are the number of studies identified and screened at each stage plus corresponding exclusion criteria, and the final number of studies included.

We screened 1,866 titles and abstracts, of which 431 papers were deemed potentially relevant and were fully reviewed. After full text review we included a final set of 128 studies, published between 2003 and 2017, reporting 179 relevant contrasts (165 contrasts reporting 1915 foci of increases in activation when contrasting empathy > baseline/neutral; only 14 contrasts reported 48 foci of decreases in activation when contrasting empathy > baseline/neutral). Figure 1 provides a full overview of the screening and selection process. From the final included contrasts on activation increases, there were 72 on empathy for pain and 89 on empathy for non-pain affective states (60 assessed empathy for negative valence emotional/sensory states, 8 positive valence emotional/sensory states and 23 mixed valence/unspecified states). Across all empathy contrasts, 99 used a cognitive/evaluative paradigm and 64 used an affective/perceptual paradigm. A chi-square test showed that the proportion of pain and non-pain paradigms was not different across these two types of paradigms [$\chi_{\left(1,\text{n}=159\right)}^{2}$ = 0.05, *p* = 0.82]. Across empathy for pain contrasts only, 44 used a cognitive/evaluative task, 27 used an affective/perceptual task^2^; 48 used an acute pain infliction paradigm, and 22 used facial pain expressions^3^. A chi-square test showed that the proportion of cognitive/evaluative and perceptual/affective paradigms was not different across the two types of stimuli [$\chi_{\left(1,\text{n}=70\right)}^{2}$ = 0.18, *p* = 0.68]. From the final included contrasts on activation decreases, there were 5 on empathy for pain and 8 on empathy for non-pain affective states. Due to the small number of reported decreases, we examined convergence across all empathy-related studies and did not perform contrasts. A full overview and characteristics of the studies can be found in Table S1.

### Convergence across all empathy-relevant studies

**Figure 2:**
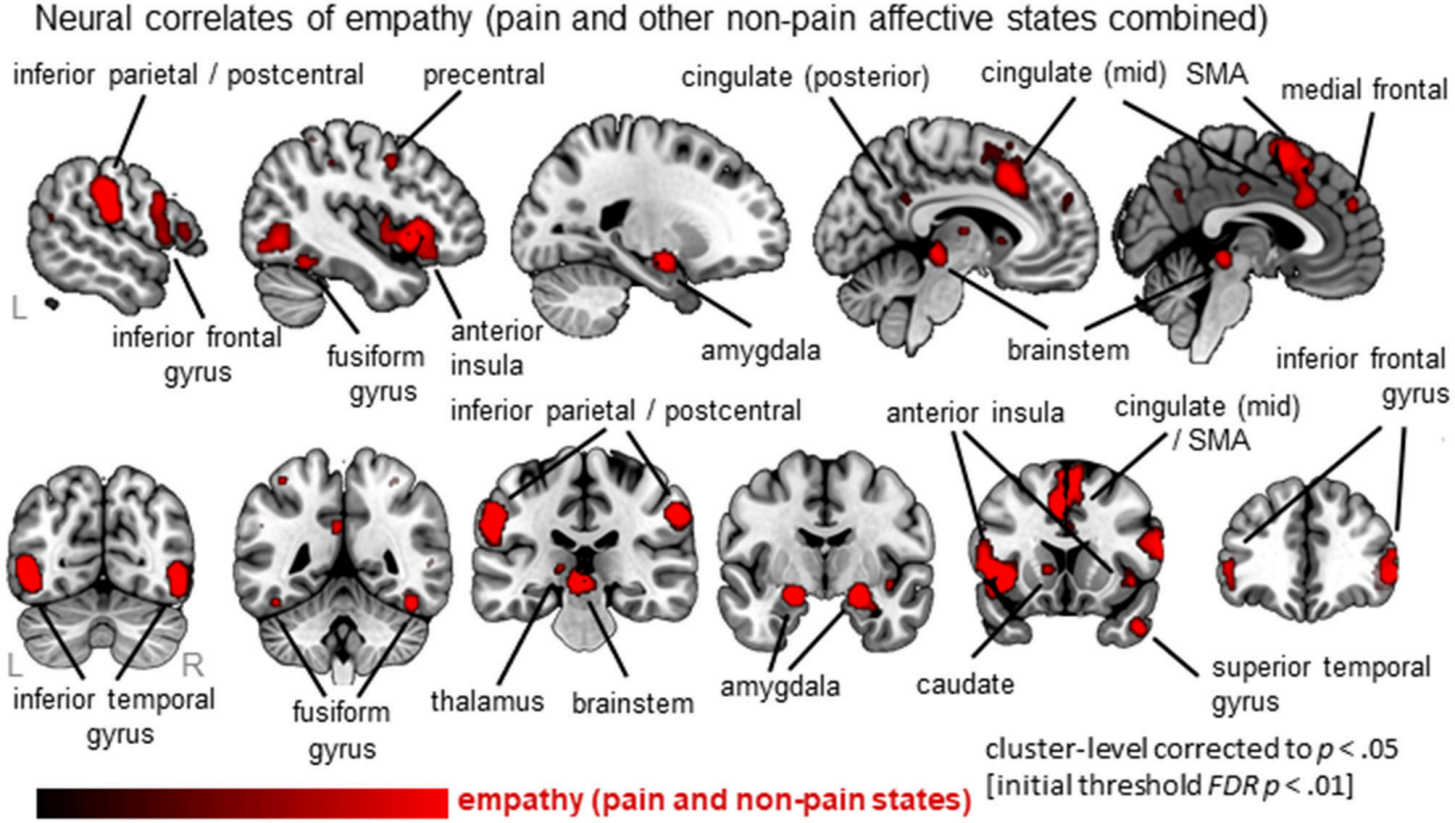
Overview of results from the ALE meta-analysis of empathy studies. The clusters show the convergence across all empathy studies (pain and non-pain; red overlay on template brain). Regions are labeled for orientation purposes.

Thirty-six clusters were identified using the ALE analysis that showed increased activation for an empathy condition compared to a baseline/neutral condition (Figure 2; Table S2; data available as a mask file upon request). The largest clusters were located in bilateral anterior insula (AI; extending to inferior frontal gyrus), the mid-cingulate cortex (MCC; also part of the medial frontal gyrus, extending to Brodmann Area 6 or the Supplementary Motor Area/SMA), and the inferior frontal gyrus (IFG). In addition, the postcentral gyrus (or primary somatosensory cortex, SI), brainstem (at level of midbrain), amygdala, inferior parietal lobe (IPL), and more posterior parts of the cingulate cortex were significantly activated. For a complete list of clusters, see Table S2.

With respect to decreased activation or an empathy condition compared to a baseline/neutral condition, no significant clusters were identified using the ALE analysis.

### Comparison between empathy for pain and empathy for non-pain negative affective states

**Figure 3:**
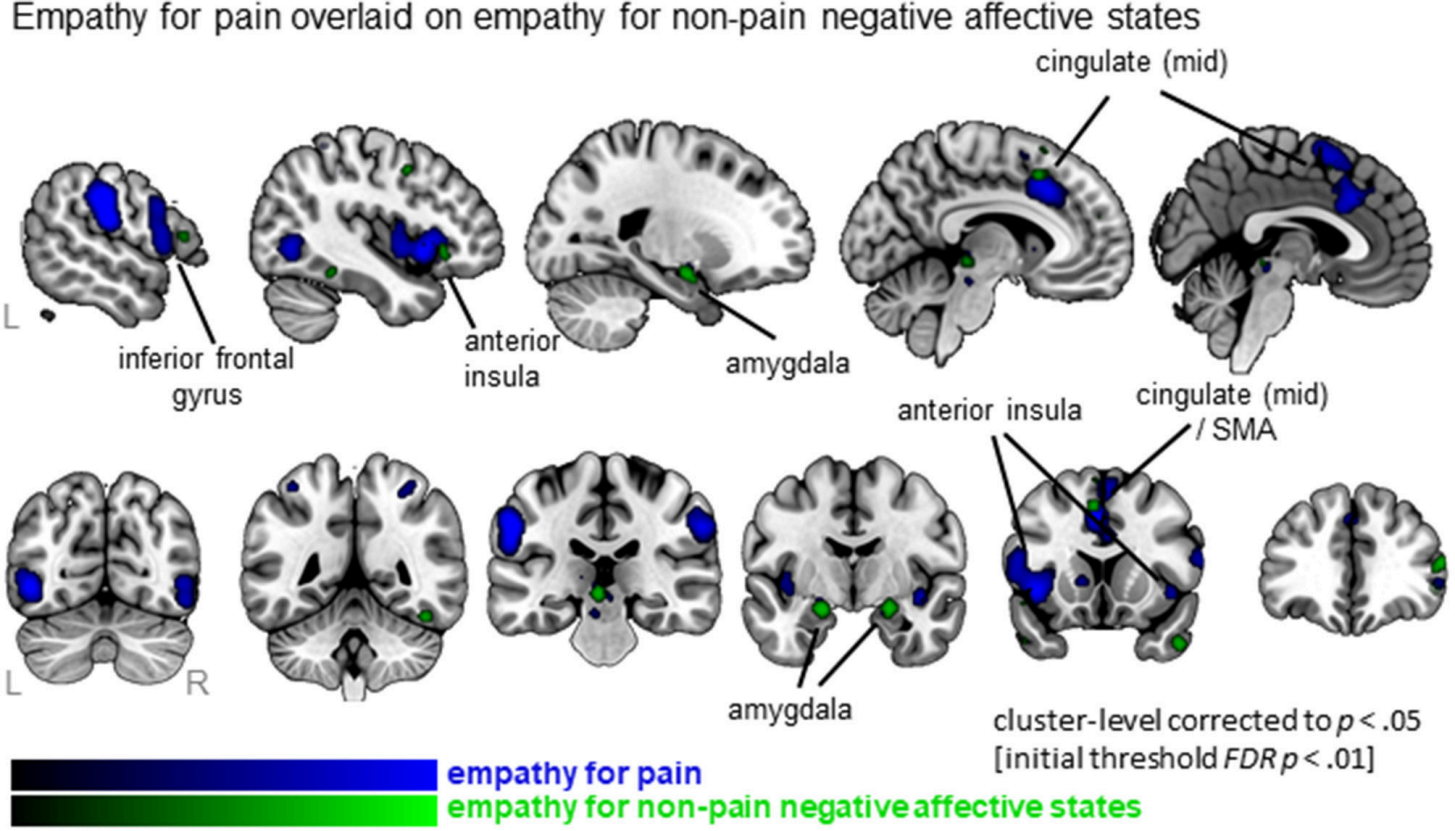
Clusters showing convergence for empathy for pain studies (blue overlay) with the clusters for empathy for non-pain negative affective states overlaid (green overlay). Labeled are regions showing the overlap in neural correlates across the two types of affective/sensory states.

When examining empathy for pain specifically, 27 clusters were identified with the largest clusters including left AI (extending to IFG), right mid insula, MCC (extending to SMA) and postcentral gyrus (SI) (see Figure 3, Table S3; data available as a mask file upon request). For empathy for non-pain negative affective states, 30 clusters were identified, including similar regions. The conjunction analysis confirmed that several regions were activated for both empathy for pain as well as empathy for non-pain negative affective states, namely the left MCC, left AI, right IFG, right superior frontal gyrus (SFG), left precentral gyrus (or primary motor area, MI), thalamus, globus pallidus, and the amygdala. When inspecting differences, however, the ALE analysis identified regions that were activated more by empathy for pain compared to empathy for non-pain negative affective states, including bilateral anterior/mid insula, MCC, medial frontal gyrus (supplementary motor area, SMA), postcentral gyrus (SI) and precuneus. No regions were identified that were more activated for empathy for non-pain negative affective states than for empathy for pain. See Table S3 for a complete list of commonalities and differences.

### Comparison between paradigms using acute pain inflictions vs. facial expressions

**Figure 4:**
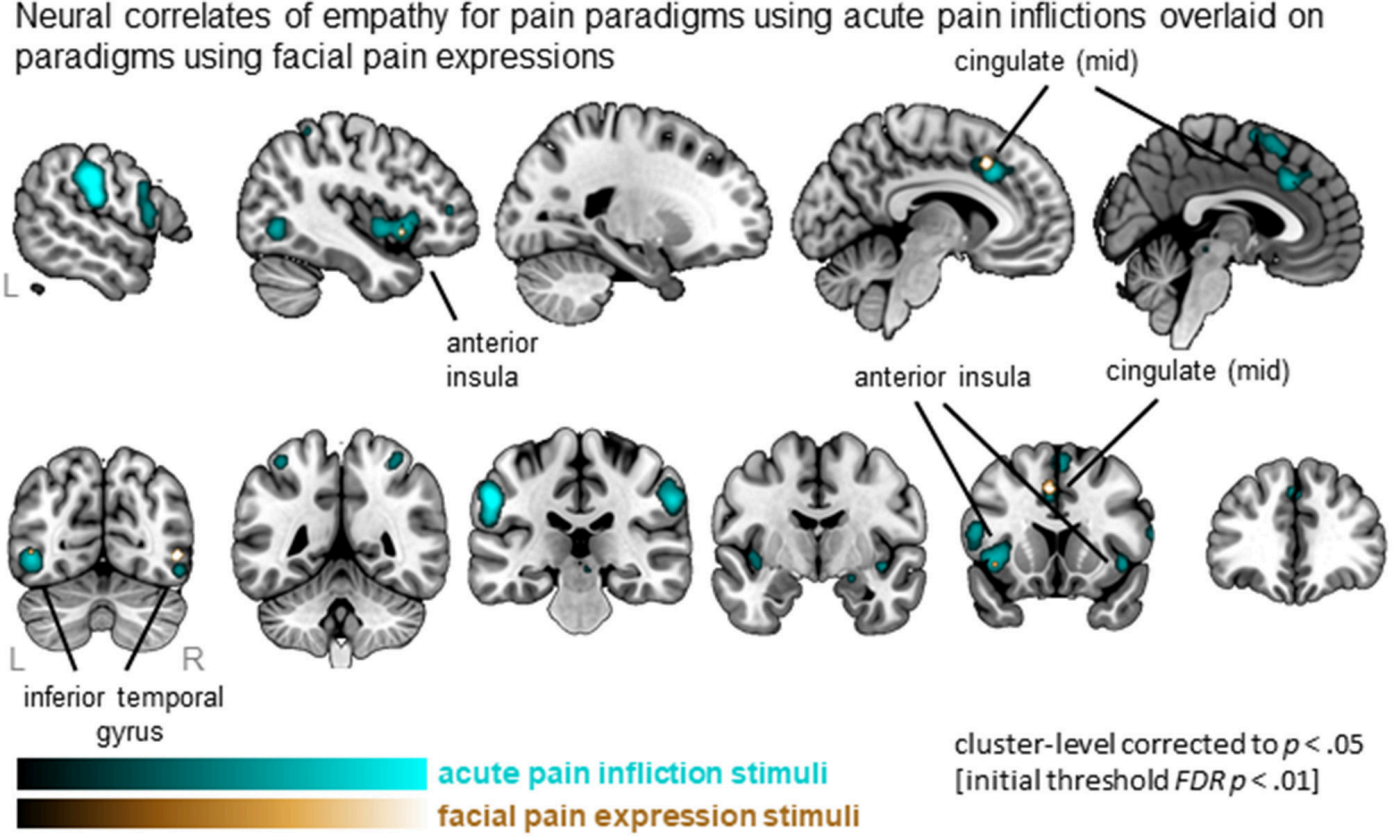
Clusters showing convergence across empathy for pain studies using acute pain infliction paradigms (cyan overlay) and studies using facial pain expression paradigms (gold overlay). Labeled are regions showing the overlap in neural correlates across the two types of stimuli.

Synthesizing data from studies using acute pain inflictions showed a total of 23 clusters that are widely distributed and include MCC, AI (extending to IFG), SMA, IFG as well as parietal and occipital regions. In contrast, four clusters were identified from the studies using facial expressions, including left MCC, left AI and bilateral inferior temporal regions (Figure 4). All 4 clusters were activated by both types of stimuli, as indicated by the conjunction analysis. Subtracting studies using facial expressions from acute pain infliction stimuli, hence, did not result in any significant clusters. The reverse, however, resulted in five clusters including medial frontal gyrus as well as clusters in the parietal lobe. See Table S4 for full details.

### Comparison between cognitive/evaluative and affective/perceptual paradigms

#### All empathy studies (pain and non-pain)

**Figure 5:**
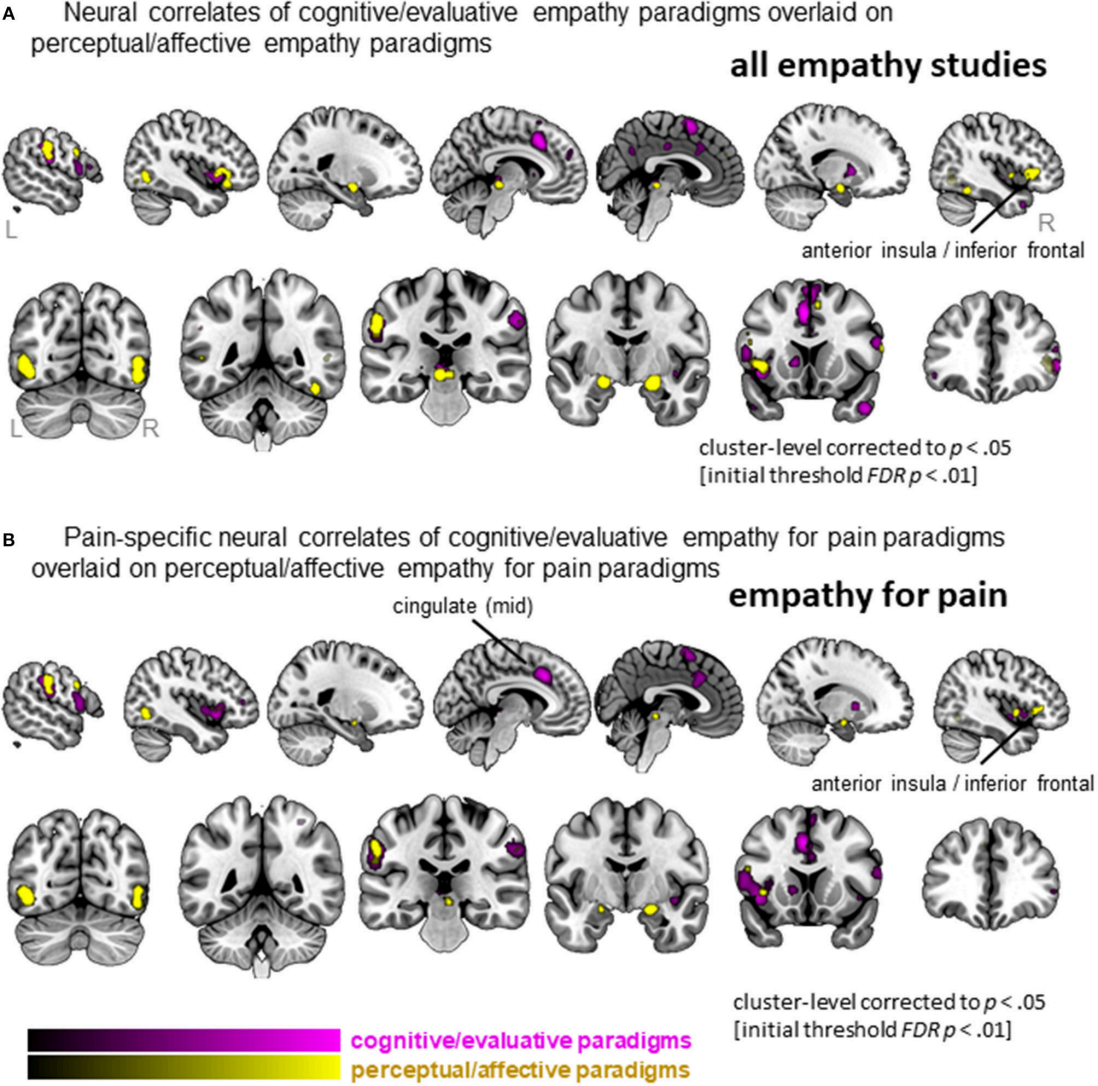
Clusters showing convergence across empathy for pain studies using a cognitive/evaluative paradigm (magenta) and studies using a perceptual/affective paradigm (yellow). **(A)** Results when all studies are included in the ALE analysis (pain and non-pain). **(B)** Results when only empathy for pain studies are included in the ALE analysis. Labeled are regions showing differences in neural correlates across the two types of paradigms.

When separating the more active cognitive/evaluative paradigms from the more passive perceptual/affective paradigms to elicit empathy, we found that 41 clusters were activated for cognitive/evaluative paradigms while 25 clusters were activated for perceptual/affective paradigms (Figure 5A, Table S5). Regions that were activated by both types of paradigm included medial frontal cortex as well as middle and inferior frontal gyri, left AI and bilateral amygdala. No clusters that were more activated by cognitive/evaluative paradigms compared to perceptual/affective were revealed. The reverse contrast, however, showed that right IFG/AI was more activated by perceptual/affective compared to cognitive/evaluative paradigms.

#### Empathy for pain

When confining the studies to empathy for pain, we found 19 clusters were activated by the more active cognitive/evaluative paradigms and 15 clusters were activated by the more passive perceptual/affective paradigms (Figure 5B, Table S6). The conjunction analysis showed that regions including the IFG, mid-insula and IPL were activated by both types of paradigms. The left mid-cingulate was found to be more activated by cognitive/evaluative paradigms, while the right IFG/AI was found to be more activated by perceptual/affective paradigms.

## Discussion

Being empathic to the felt experiences of others increases social connection and promotes prosocial behavior, facilitating smoother interactions in the social world (Davis, 1994; Preston and De Waal, 2002; Singer and Lamm, 2009; Zaki, 2014; Decety et al., 2016). Pain is a core component of human suffering, hence, experimental studies in which the participant observes another person in pain provide a powerful way to investigate the behavioral and neural correlates of empathy. Whether neural correlates of empathy for pain are unique compared to those of empathy for other non-pain negative affective states, however, has not yet been investigated. Here, we performed a meta-analysis of functional brain imaging studies of empathy to both replicate previous findings of a core empathy neural network and to identify whether neural correlates of empathy for pain are unique and distinct from those of empathy for non-pain negative affective states. In addition, we aimed to identify commonalities and differences between stimuli and paradigms to evoke empathy for pain. This information is particularly relevant for studying empathic responses in different contexts, with different target affective/sensory states and with different populations. One specific example would be when studying empathy in the context of chronic pain instead of acute pain, where given the complexity and contextual relevance of the pain, an acute pain infliction paradigm may be insufficient to capture the full empathic response to pain. The results from this meta-analysis will hence inform the design of experimental studies of evoked pain-related empathy across different contexts.

### Core empathy network

In line with previous findings, we identified a core neural network of empathy. When synthesizing all empathy studies (pain and non-pain positive and negative affective states), the bilateral anterior insula (AI, extending to the inferior frontal gyrus, IFG), bilateral mid-cingulate cortex (MCC, extending to the supplementary motor area, SMA), postcentral gyrus (or primary somatosensory cortex, SI), inferior parietal lobe (IPL), thalamus, amygdala, brainstem (midbrain) emerged as core regions. These brain regions have all been identified by previous meta-analyses performed in this field (Fan et al., 2011; Lamm et al., 2011; Bzdok et al., 2012). As these extant meta-analyses have used different parameters, our replication here demonstrates the robust involvement of these core brain regions in empathic responses.

### Unique aspects to neural correlates of empathy for pain

When synthesizing studies of empathy for pain only, a strikingly similar network emerged. Moreover, conjunction analyses confirmed extensive overlap between neural circuitry of empathy for pain and empathy for non-pain negative affective states. In addition to inferior and superior frontal regions, thalamus, globus pallidus, and amygdala, the left MCC and left AI were identified as common activated regions. Within the empathy for pain literature, these latter two regions have gained considerable attention and have been consistently identified as a prominent point of convergence (see e.g., Corradi-Dell'acqua et al., 2011; Lamm et al., 2011; Zaki et al., 2016). Moreover, these regions are also robustly activated by the physical experience of pain (Peyron et al., 2000; Wager et al., 2013; Zaki et al., 2016), and this neural overlap has been taken as support for the so-called “shared representation” account (Lamm et al., 2011). This model proposes shared representations between self and other, indicating that neural circuits involved in the personal experience of a certain state also underlie the understanding and sharing of that state when observing it in others (Decety and Sommerville, 2003; Keysers and Gazzola, 2006). This shared representation has been observed in the AI in non-pain negative affective states as well (see e.g., Wicker et al., 2003; Lamm et al., 2015). In line with that, our data show convergence in these core regions (i.e., AI and MCC) -at least the left homologs- for observing non-pain negative affective states. It would be interesting for future studies to incorporate direct comparisons of experiencing pain and observing pain in contrast to experiencing and observing non-pain affective states. It might be that the neural responses to observed pain as well as responses to experienced pain reflect emotional rather than pain-specific responses. Indeed, the MCC and AI are most prominently involved in the affective aspects of pain (Villemure and Bushnell, 2009; Wiech et al., 2010; Schweisfurth et al., 2015) rather than in any sensory aspects (i.e., more posterior parts of the cingulate and insular cortex are believed to be involved in sensory aspects of pain) (Garcia-Larrea and Peyron, 2013). Further, these regions are involved in much more than only processing of experienced and observed pain. It is believed that the MCC (extending into SMA) and AI (extending into IFG) form a network that is critically associated with the representation of one's own and the other person's emotional states, as well as interoceptive awareness (Critchley et al., 2004; Craig, 2009). Relatedly, it has been suggested that the shared representation may be due to shared saliency of the stimuli (Valentini and Koch, 2012), and indeed, in resting state fMRI these regions are commonly found to be co-activated, referred to as the “salience network,” having a central role in the detection of behaviorally relevant and hence salient stimuli (Uddin, 2015).

In addition to commonalities, important differences emerged when contrasting empathic responses for pain vs. non-pain negative affective states. In particular, empathy for pain recruited a number of additional brain regions compared to those recruited by empathy for non-pain negative affective states. These included bilateral mid-insula, which is in line with previous studies showing that while anterior insula responded to both empathy for pain and non-pain stimuli (i.e., negative or aversive stimuli in general), mid-insula responded more specifically to empathy for pain (Corradi-Dell'acqua et al., 2011). Furthermore, empathy for pain recruited more extensive parts of the MCC as well. One potential interpretation of these findings is that pain evokes a stronger empathic response compared to other negative states (e.g., anger, sadness, fear, disgust, distress). Also when comparing experienced vs. observed pain, studies have found that experiencing pain activates more extensive regions (with a posterior gradient) compared to merely observing pain (Lamm et al., 2011). This may indicate an anterior to posterior gradient -in both core empathy regions AI and MCC- that shifts according to whether the person is observing non-pain negative states, observing pain states, or experiencing pain. In addition, empathy for pain vs. non-pain negative affective states recruited more brain regions that are involved in the experience of pain (e.g., pre- and postcentral gyrus) and preparing motor responses in response to pain (Morrison et al., 2007). These findings are somewhat in contrast with findings of Lamm et al. (2011), who identified that the experience of pain, but not observing another in pain, evoked neural activation in more posterior parts of the insula and in more posterior and superior parts of the cingulate cortex. This difference might be due to our broader definition of empathy, or that our more recent search resulted in more than double the number of included studies (we included 72 studies; Lamm et al. included 32 studies). Also of note, our analyses comprised more studies of empathy for pain (72 contrasts, 874 foci) compared to empathy for non-pain negative affective states (60 contrasts, 678 foci), which may have contributed to our findings of differences across the two domains. At the same time, given the differences between the analyses (e.g., image-based analysis of Lamm et al. vs. our coordinate-based analysis) it is notably challenging, if not invalid, to compare the localization of the clusters between meta-analyses and to interpret any visual differences.

### Influence of type of stimuli and paradigm to evoke empathy for pain

When comparing experimental stimuli to evoke empathy for pain, we identified that both acute pain inflictions and facial pain expressions activated the core empathy regions (i.e., left MCC, left AI). Acute pain inflictions additionally recruited other parts of left medial frontal lobe, as well as bilateral postcentral gyrus (SI)/inferior parietal lobe (IPL), when compared to facial expressions. The involvement of SI/IPL might reflect processes involved in prediction and anticipation of what would happen in acute pain infliction situations, as these stimuli require inferences about how the other person feels upon receiving the pain induction. It has been proposed that the combined activation of SI/IPL and IFG regions underlies action observation and action understanding (Rizzolatti et al., 2014). It was somewhat unexpected that the contrast between acute pain inflictions and facial pain expressions did not point toward more primary motor areas (e.g., middle frontal gyrus/premotor area or precentral gyrus). Also surprisingly, facial pain expressions did not activate more occipital regions involved in the processing of faces more generally (e.g., fusiform face area). This is somewhat surprising given that the acute pain infliction stimuli depict non-face body parts. Both may be due to the relatively small number of included studies in the contrast (i.e., 22 contrasts reported on a total of 260 foci, compared to 48 contrasts and 605 foci in the acute pain infliction paradigms) and hence lower power for this comparison. Nevertheless, given that the number of contrasts is rather small, the results are quite robust: both the ALE analysis of studies using painful faces as well as the conjunction with studies using acute pain infliction reveal activity in the core empathy network. Hence, the data suggest that both facial expression and pain infliction stimuli yield robust empathy-related neural activation patterns.

Interestingly, our ALE results for studies using pain face stimuli are somewhat different to the findings of a recent meta-analysis that examined empathy for (non-pain) emotional faces (Del Casale et al., 2017). While we found left MCC, left AI, and bilateral inferior temporal gyrus activation in response to viewing pain faces, Del Casale et al. identified a network encompassing left anterior cingulate, posterior cingulate, right insula, amygdala, putamen, precentral gyrus (MI), and superior frontal gyrus in response to emotional non-pain faces. Although no formal comparison has been made, there seems to be little overlap in findings. One potential explanation is that observing a facial pain expression is more ambiguous compared to other affective states (i.e., pain expressions are generally most difficult to identify and are accompanied by the lowest confidence ratings; Kappesser and Williams, 2002), which in turn may contribute to heightened levels of threat perception. Additionally, observing a pain expression might potentially be more relevant for the observer him/herself compared to a non-pain emotional face as it could signal an immediate physical threat for the self as well. Alternatively, methodological aspects might explain the difference, as Del Casale et al. also included emotional faces with a positive valence, while pain has an inherently negative valence. In any case, it would be interesting to do a direct comparison of non-pain negative facial expressions vs. facial pain expressions.

In addition to type of stimulus, we characterized studies based on their instructions: whether they gave active instructions to engage empathy processes (i.e., cognitive/evaluative paradigms) or more passive instructions without explicit directions (i.e., perceptual/affective paradigms). A previous meta-analysis revealed that while both types of paradigms elicit left AI, perceptual/affective empathy paradigms tend to elicit more right AI/IFG activation, while cognitive/evaluative paradigms tend to elicit more MCC activation (Fan et al., 2011). Here, we replicate this partially, showing that left AI was elicited by both types of paradigms, while perceptual/affective paradigms activated more right IFG/AI (but we did not find that MCC was more activated by cognitive/evaluative paradigms). When specifically investigating the effect of paradigm in the study of empathy for pain, the findings are completely in line with the meta-analysis of Fan et al. (2011). When focusing on empathy-for-pain studies, both paradigms elicited left IFG/AI, while perceptual/affective paradigms activated more right IFG/AI, and cognitive/evaluative paradigms activated more left MCC. Hence, the AI shows a particularly pronounced asymmetry in its involvement with empathy for pain and empathy for non-pain states: right IFG/AI is more involved in uninstructed empathic responses, while left AI is involved in both types of paradigms (instructed and uninstructed). The left AI is also robustly activated by different types of stimuli, as noted above (acute pain infliction vs. pain faces). Previous studies have also highlighted differences between left and right AI in terms of function and anatomy (Craig, 2009). There are many tasks and emotions that activate either unilateral or bilateral AI, and the general trend seems to be that stimuli that activate the right AI are more arousing or “energy consuming” (see e.g., Craig, 2009, 2011; for an overview). This might indicate that uninstructed empathy paradigms elicit more arousal, but further studies are needed to explore this and explain the differences in left vs. right AI in the context of empathy.

### Considerations and future steps

This meta-analysis summarizes available neuroimaging data pertaining to the question of whether or not neural correlates of pain-related empathy is unique in comparison to those of empathy for other non-pain negative affective states. In a meta-analysis, it is often implicitly assumed that the presence or absence of neural overlap provides compelling evidence for answering this question. However, a thorough consideration of this question must involve integration of both neuroimaging and behavioral data, as well as theoretical development. Advances in social theories of pain provide some indication as to *why* empathy for pain may involve different psychological and neural processes compared to empathy for non-pain negative affective states. Recent theoretical models of the social context of pain (Karos et al., 2018) and of parent-child interactions in pain (Simons et al., 2016) propose that observing another in pain can constitute an immediate physical threat—not only for the person who experiences pain, but also to the observer him/herself. Seeing someone else in pain could indicate that there is a nearby aggressor who could also cause harm in the observer. Likewise, it could indicate that there is a naturally occurring physical stimulus or event (e.g., broken glass on the floor or an earthquake) that could lead to bodily harm in the observer. Thus, when we observe another person in pain it may be biologically adaptive to infer a potential immediate physical threat to the self. This concurrent threat to the observed and the observer is relevant for some non-pain affective states such as fear, but less clear for other negative affective states such as sadness, in which we often assume a more distal or personal source of emotion in the observed individual. Future studies investigating the role of self-directed protective cognitions, behaviors, and neural responses (e.g., in motor areas involved in preparing for action) in pain and non-pain empathy studies would be particularly informative.

Some inherent limitations to meta-analyses should be noted. First, we included only published studies, making our results vulnerable to publication bias. In addition, we cannot exclude the possibility that we missed studies that have examined identical or similar processes as were included in this meta-analysis (e.g., neural responses to observing sad faces), but have not examined their results in the context of empathy, and thus were not identified by our search. Our search terms, however, were designed carefully to be sensitive to identify relevant studies. Furthermore, to avoid potential bias, we did not include studies that did not present whole brain analyses (e.g., only focused on one region of interest) or that did not present a contrast between an empathy condition and baseline or neutral condition (e.g., studies that presented group differences only, correlation analyses, or a contrast between two empathy conditions). We cannot exclude the possibility, however, that excluding these studies may have biased our results. Future studies could consider collecting original data from the authors to avoid this type of bias. We should furthermore be mindful that the comparison of studies is confounded by the statistical strategies the individual studies adopted (e.g., differences in statistical thresholds and chosen cluster-extent thresholds). In addition, some studies have compared an empathy condition to fixation or rest, while others compared to a neutral condition (e.g., pain face vs. a neutral face). However, the large number of studies included in this meta-analysis may negate the confounding effect of variability in statistical scrutiny and contrast choices. Lastly, as already touched upon, we should keep in mind that from the presented results we can only describe convergence, as well as overlap and differences in convergence of activated neural circuitry. Our results cannot make any inferences on whether underlying mechanisms of overlapping neural circuitry are identical or whether supported functions are similar. Additional research incorporating behavioral and/or physiological measures is necessary to advance our understanding on the functional relevance of identified core brain areas in different types of empathy.

## Conclusions

The current ALE meta-analysis of functional imaging studies on empathy shows, once again, that there is a core neural network for empathy, comprising the anterior insula (AI) and mid-cingulate cortex (MCC). These regions are recruited robustly, irrespective of the affective state of the observed (i.e., pain and non-pain negative affective states), of the type of stimuli (i.e., acute pain infliction and painful faces), and of the instructions (i.e., actively engaging empathic responses or more automated processing). Hence, to some extent this allows one to tailor the paradigm and stimuli within an empathy study to a specific context (e.g., using stimuli that incorporate contextually relevant details when investigating empathic responses in chronic pain). However, these core regions do not act in isolation; rather, they interact with many other brain regions. When looking beyond these core regions, important differences are identified among the various methods to evoke and study empathy. This has important implications for the generalizability of empathy studies, as our findings indicate that results do not necessarily generalize across different contexts (i.e., affective/sensory states) and designs. In particular, our findings show that there are core regions, as well as unique regions, that are activated by different stimuli and paradigms. Hence, studies may benefit from examining pain and non-pain negative affective states separately, and from targeting the specific affective/sensory state of interest in comparison to a neutral state. Lastly, one should be aware of commonalities and differences across different states and designs, and take these into account when designing, interpreting, or trying to replicate, experimental studies on the neural correlates of empathy.

## Author contributions

IT and LS contributed conception and design of the study. IT, AP, MF, and CK performed screening and full text reviews. IT, AP, MF, and CK performed data extraction. IT, CK performed data-analysis. IT wrote manuscript. IT, AP, MF, CK, LH, JH, and LS contributed to the manuscript, read and approved the submitted version.

### Conflict of interest statement

The authors declare that the research was conducted in the absence of any commercial or financial relationships that could be construed as a potential conflict of interest.

## Footnotes

^1^Note that emotional pain was classified as non-pain (i.e., a non-pain negative affective state), and only physical pain was classified as pain.

^2^One contrast combined the cognitive/evaluative and affective/perceptual conditions, and hence was not assigned to any paradigm dataset, but was included in the empathy for pain dataset.

^3^Two contrasts used a different stimulus (written story) and hence were not assigned to any stimulus dataset, but was included in the empathy for pain dataset.
