# Supplementary material for: Is Empathy for Pain Unique in Its Neural Correlates? A Meta-Analysis of Neuroimaging Studies of Empathy
Source: Front Behav Neurosci. 2018 Nov 27;12:289. doi: 10.3389/fnbeh.2018.00289 (PMC6277791; doi:10.3389/fnbeh.2018.00289)
Supplement: Supplementary file 1 [file Table_1.pdf]

TABLE S1 | Overview and characteristics of all studies included in the meta-analysis

Paper	Field strength	Participants (Female)	Smoothing level (mm)	Type of stimuli	Affective / sensory state targeted	Cognitive/evaluative or affective/perceptual	If C/E: task	Relevant contrast(s)
Akitsuki et al., 2009	3T	26 (14F)	6	Pain infliction to body part (animated)	Pain	A/P		Pain > no pain
Arbuckle et al., 2017	3T	26 (1F)	9	Pain infliction to body part (animated)	Pain	C/E	Rate pain experienced by other person	Pain > no pain
Azevedo et al., 2013	3T	27 (16F)	8	Pain infliction to body part (animated)	Pain	A/P		Pain > no pain
Azevedo et al., 2014	3T	12 (12F)	8	Pain infliction to body part (animated) plus facial pain expression	Pain	A/P		Pain > no pain
Aziz-Zedah et al., 2012	3T	13 (13F)	7.5	Pain infliction to body part (animated)	Pain	A/P		Pain > no pain
Bagozzi et al., 2013	3T	24 (8F)	8	Facial expressions (photos)	Anger, disgust, happiness, surprise	A/P		Negative (angry, disgust) > neutral<br>Positive (happy, surprised) > neutral
Benuzzi et al., 2008	3T	15 (15F)	6x6x9	Pain infliction to body part (animated), as well as disgusting stimulus	Pain, disgust	C/E	Rate unpleasantness of videos	Pain > neutral<br>Disgust > neutral
Berlingeri et al., 2016	1.5T	25 (13F)	8	Pain infliction to body part (animated)	Pain	C/E	Rate pain experienced by other person	Pain > no pain
Bodden et al., 2013	1.5T	30 (15F)	8	Drawings of faces	Undefined	C/E	Affective ToM: complete sentences by inferring affect of face	Emotional > control (physical causality inference)
Bos et al., 2015	3T	24 (0F)	8	Pain infliction to body part (animated)	Pain	A/P		Pain > no pain

Botvinick et al., 2005	1.5 T	12 (12F)	12	Facial expressions (video clips)	Pain	A/P		Pain > neutral
Bruneau et al., 2012	3T	49 (18F)	5	Written scenarios	Pain, emotional pain (suffering)	C/E	Rate compassion felt for the protagonist	Pain > neutral<br>Emotional pain > neutral
Bruneau et al., 2012	3T	41 (25F)	5	Written scenarios	Pain, emotional pain (suffering)	C/E	Rate pain intensity or actively empathize (results are collapsed across instructions)	Pain > neutral<br>Emotional pain > neutral
Brunnlieb et al., 2013	3T	18 (0F)	8	Drawings	Anger, sadness, pain, anxiety	C/E	Imagine how they would feel	Negative emotion/pain > neutral
Budell et al., 2010	3T	18 (9F)	8	Facial expressions (video clips)	Pain	C/E	Rate pain intensity	Pain > no pain
Cheng et al., 2007	3T	14 (7F)	8	Pain infliction to body part (animated)	Pain	A/P		Pain > no pain
Cheng et al., 2010	3T	36 (18F)	8	Pain infliction to body part (animated)	Pain	C/E	Observe scenes with perspective of loved one or stranger	Pain loved one > no pain loved one<br>Pain stranger > no pain stranger
Chiao et al., 2009	NR	14 (14F)	8	Natural scenes (faces plus context)	Emotional pain (suffering)	A/P		Emotional Pain > no pain
Cheon et al., 2013	3T	27 (5F)	8	Natural scenes (faces plus context)	Emotional pain (suffering)	A/P		Emotional Pain > no pain
Choe et al., 2015	3T	178 (0F)	6	Facial expressions (photos)	Fear, anger	C/E	Select the face identical to the target face	Fear > control<br>Anger > control
Coll et al., 2017	3T	30 (20 F)	8	Facial expressions (video clips)	Pain	A/P		Pain > no pain
Corradi-Dell'Acqua et al., 2011	3T	28 (28F)	8	Pain infliction to body part (photos)	Pain	A/P		Pain > no pain
Danziger et al., 2009	1.5T	13 (7F)	8	Pain infliction to body part (cue; photos) plus facial expressions (photos)	Pain	C/E	Imagine how the other person feels	Pain > no pain (cue)<br>Pain > no pain (face)
Dapretto et al., 2006	3T	10 (1F)	AIR	Facial expressions (photos)	Anger, fear, happiness, neutrality, sadness	A/P		Emotional > baseline
De Greck et al., 2012a	1.5T	20 (12F)	6	Facial expressions (photos)	Anger, disgust, happy, neutral	C/E	Empathize with person in photo	Anger > control<br>Disgust > control<br>Joy > control

De Greck et al., 2012b	3T	20 (11F)	8	Facial expressions (photos)	Anger	C/E	Empathize with person in photo	Anger > baseline
Decety et al., 2008	3T	17 (9F)	6	Pain infliction to body part (animated)	Pain	A/P		Pain > no pain
Decety et al., 2009	3T	8	6	Pain infliction to body part (animated)	Pain	A/P		Pain > no pain
Decety et al., 2010	3T	57 (28F)	6	Pain infliction to body part (animated)	Pain	A/P		Pain > no pain
Deeley et al., 2006	1.5T	9 (0F)	NR	Facial expressions (photos)	Fear	A/P		Fear > neutral<br>*Neutral > fear*
De Gelder et al., 2004	3T	7 (3F)	6	Emotionally expressive gestures (photos)	Fear, happy	A/P		Fear > neutral<br>Happy > neutral
Dong et al., 2016	3T	36 (0F)	6	Facial expressions (photos)	Pain	C/E	Evaluate whether person is experiencing pain	Pain > no pain
Enzi et al., 2016	3T	20 (0F)	8	Pain infliction to body part (animated)	Pain	C/E	Evaluate whether person is experiencing pain	Pain > no pain
Ernst et al., 2013	3T	18 (12F)	8	Facial expressions (photos)	Happiness, sadness, disgust, fear, surprise, anger, contempt	A/P		Emotional (all together) > control
Fan et al., 2014	3T	21 (0 F)	10	Pain infliction to body part (photos)	Pain	C/E	Evaluate whether photo depicts pain	Pain > no pain
Feng et al., 2016	3T	22 (11F)	5	Pain infliction to body part (including face; photo)	Pain	C/E	Empathize and rate how much pain other person experiences	Pain > no pain<br>*No pain > pain*
Fourie et al., 2017	3T	38 (21F)	NR	Facial expressions (video clips)	Pain, emotional pain (distress)	C/E	Empathize with person in video clip and for emotional pain: rate their empathic concern	Pain > no pain<br>Social pain > no pain
Fujino et al., 2014	3T	11 (9F)	NR	Pain infliction to body part (animated)	Pain	A/P		Pain > no pain
Gao et al., 2017	3T	35 (0F)	6	Pain infliction to body part (photos)	Pain	C/E	Rate how much pain other person experiences	Pain > no pain

Geday et al., 2003	PET	9 (0F)	NR	Facial expressions and complex social scenes (photos)	Sadness, disgust (unpleasant)	A/P		Unpleasant > neutral<br>*Neutral > unpleasant*
Geday et al., 2007	PET	14 (7F)	12	Faces plus context (photos)	Positive and negative	A/P		Emotional (taken together) > neutral
Göttlich et al., 2017	3T	26 (20 F)	8	Drawings	Distress	A/P		Distress > neutral
Greimel et al., 2010	1.5T	47 (0 F)	8	Facial expressions (photos)	Happy, sad, neutral	C/E	Infer emotional state of other and evaluate own state (self)	Emotional (other) > neutral<br>Emotional (self) > neutral
Grice-Jackson et al., 2017	1.5T	21<br>13<br><br>10	8	Pain infliction to body part (photo)	Pain	C/E	Evaluate whether they experience any pain while observing	Pain > no pain (control)<br>Pain > no pain (sensory responders)<br>Pain > no pain (affective responders)
Grosbras et al., 2006	1.5T	20 (10F)	6	Facial expressions, hand movements (video clips)	Anger	A/P		Angry > control (face)<br>Angry > control (hand)
Gu et al., 2010	3T	18 (9F)	8	Pain infliction to body part (photo)	Pain	Both	Evaluate whether another is in pain or not; or evaluate laterality	Pain > no pain
Gu et al., 2013	3T	18 (9F)	8	Pain infliction to body part (photo)	Pain	C/E	Evaluate whether other person experiences pain or evaluate laterality	Pain (instructions collapsed) > no pain<br>*No pain > pain*
Hadjikhani et al., 2014	3T	31 (3 F)	8	Facial expressions (photos)	Pain	A/P		Pain > no pain
Han et al., 2009	3T	24 (12F), 22 (11F)	8	Pain infliction to body part (photos)	Pain	A/P		Pain > no pain (group 1)<br>Pain > no pain (group 2)
Han et al., 2017	3T	33 (16F)	8	Pain infliction to body part (videos)	Pain	A/P		Pain > no pain (passive)<br>Pain > no pain (button press)
Horan et al., 2014	3T	23 (16F)	6	Facial expressions (photos)	Happy, Sad, Angry, and Afraid	A/P		Emotional > baseline
Jackson et al., 2017	4T	51 (51F)	8	Facial expressions (video clips)	Pain	C/E	Rate how much pain other person experiences	Infant pain > no pain<br>Adult pain > no pain<br>Adult pain > no pain (nurses)
Jankowiak-Siuda et al., 2015	3T	27 (14F)	8	Facial expressions plus pain infliction (series of photos)	Pain	C/E	Rate how much compassion they feel for other person	Pain > no pain

Jensen et al., 2014	3T	18 (10 F)	8	Real life (participant sees face of patient and the heat thermode)	Pain	A/P		Pain > no pain<br>No pain > pain
Kana et al., 2016	3T	15	8	Emotional facial/body expressions in social interactions	Happy, Sad, Angry, and Afraid	C/E	Choose correct emotion of blurred face in interaction	Emotional (all collapsed) > neutral
Kanske et al., 2015	3T	25 (14F)<br>178 (102F)	8	Facial expressions (video clips)	Negative emotions	C/E	Rate how they feel and how much compassion they feel	Negative emotion > neutral<br>Negative emotion > neutral
Kim et al., 2009a	3T	21 (12 F)	7	Facial expressions (photos)	Sad, neutral	Both	View passively or with an active compassionate attitude	Sad (collapsed both instructions) > neutral
Kim et al., 2009b	1.5T	14 (6F)	9	Facial expressions and emotional story (video clips)	Angry, happy	C/E	Make inferences about what happened (given the emotional cues)	Angry > control<br>Happy > control
Kim et al., 2010	3T	19 (0 F)	8	Drawings	Not specified	C/E	Make inference about what characters feels	Empathy > control (physical causality inference)
Kim et al., 2011	3T	19 (0 F)	8	Emotional scenes (IAPS)	Threats (of natural and artificial origin)	C/E	Match the target to two choice stimuli	Negative emotion > control
Klimecki et al., 2013	3T	94 (94F)	6	Emotional scenes (video clips)	Distress	C/E	Rate how much empathy, positive and negative affect they experienced	High distress > control<br>*Control > high distress*
Krach et al., 2015	3T	16 (0F)	8	Pain infliction to body part (photos)<br>Drawings	Pain, social pain (embarrassment)	C/E	Evaluate how much pain other person experiences<br>Evaluate level of vicarious embarrassment	Pain > no pain<br>Social pain > no pain
Kramer et al., 2010	3T	16 (10F)	8	Drawings	Anger, sadness, pain, anxiety	A/P		Emotional > neutral<br>*Neutral > emotional*
Labek et al., 2017	3T	17 (14 F)	8	Drawings	Negative (mourning)	A/P		Negative emotion > control
Lamm et al., 2008	3T	18 (9F)	6	Pain infliction to body part (photos)	Pain	C/E	Evaluate amount of pain inflicted	Pain > no pain
Lang et al., 2011	3T	22 (13 F)	8	Auditory stimuli	Negative emotions	A/P		Negative > control<br>*Control > negative*

Lee et al., 2006	1.5T	14 (1 F)	8	Written scenarios	Distress	C/E	Answer questions on empathy-related info	Distress > neutral
Lee et al., 2010	1.5T	18 (9 F)	9	Drawings (comic strips)	Happiness, fear, anger, physical distress, pity	C/E	Choose correct ending to story	Empathy inference > control (physical causality inference)
Lee et al., 2013	3T	12 (12F)	8	Facial expressions (video clips)	Happy, sad, fearful, and neutral	A/P		Emotional > neutral
Leiberg et al., 2012	1.5T	24 (24F)	12	Emotional scenes (IAPS)	Negative	A/P		Emotional > neutral
Lenzi et al., 2013		23 (23F)	8	Facial expressions (photos)	Joy and distress	C/E	Watch or imitate and empathize with the faces	Joy/Distress (collapsed across instructions) > neutral
Luo et al., 2014	3T	36	8	Pain infliction to body part (video)	Pain	C/E	Evaluate whether other person experiences pain	Pain > no pain
Luo et al., 2015	3T	30 (A/A genotype)<br>30 (G/G genotype)	8	Pain infliction to body part (video) using two type of models	Pain	C/E	Evaluate whether other person experiences pain	Pain > no pain (A/A, model A)<br>Pain > no pain (A/A, model B)<br>Pain > no pain (G/G, model A)<br>Pain > no pain (G/G, model B)
Mascaro et al., 2014	3T	36	8	Infant crying (auditory)	Negative / Distress	A/P		Distress > control
Mathur et al., 2010	3T	28	8	Facial expressions in natural scenes (photos)	Negative	C/E	Rate the level of experienced empathy	Emotional pain > neutral
Mathur et al., 2016	3T	15	8	Facial expressions in natural scenes (photos)	Negative	C/E	Rate the level of experienced empathy	Emotional pain > neutral
Mazza et al., 2013	1.5T	10 (10F)	NR	Emotional scenes	Negative emotions	A/P		Negative emotion > control
Mazzola et al., 2010	3T	30	10	Facial expressions (photos)	Negative	A/P		Pain > no pain
Melchers et al., 2015	1.5T	60	8	Stills from a video on embarrassing scenes	Vicarious embarrassment	A/P		Vicarious embarrassment > neutral
Mercadillo et al., 2011	3T	24 (12F)	NR	Emotional scenes (photos)	Suffering	C/E	Indicate how much compassion they feel	Suffering > neutral social scenes
Mercadillo et al., 2015	3T	24 (12F)	NR	Emotional scenes (photos)	Suffering	C/E	Indicate how much compassion they feel	Suffering > neutral social scenes
Michalska et al., 2013	3T	65	6	One person hurting another (video clip)	Pain	A/P		Pain > no pain

Molenberghs et al., 2016	3T	48	6	One person hurting another (photo)	Pain	A/P		Pain > no pain
Moll et al., 2007	1.5T	12 (6F)	8	Scenarios (written)	Compassion	A/P		Compassion > neutral
Montoya et al., 2012	3T	17 (17F)	6	Crying (auditory)	Distress, happy, sad	A/P		Happy > neutral<br>*Neutral > happy*<br>Sad > neutral<br>*Neutral > sad*<br>Low distress > noise<br>*Noise > low distress*<br>High distress > noise<br>*Noise > high distress*
Morelli et al., 2014	3T	32	8	Facial/Body expression (photos)	Happy, anxiety, pain	C/E	Empathize with individuals on photos	Anxiety > neutral<br>Happy > neutral<br>Pain > no pain
Moriguchi et al., 2007	1.5T	14	6	Pain infliction to body part (photo)	Pain	C/E	Evaluate how much pain other person experiences	Pain > no pain
Morrison et al., 2004		14	5	Pain infliction to body part (photo)	Pain	A/P		Pain > rest
Morrison et al., 2007a	1.5T	12 (5F)	NR	Pain infliction to body part (photos)	Pain	A/P		Pain > no pain
Morrison et al., 2007b	1.5T	16 (8F)	6	Pain infliction to body part (photo)	Pain	C/E	Press button when nature of photo was discerned	Pain > no pain
Morrison et al., 2013	1.5T	14 (7F)	6	Pain infliction to body part (photo; grasping or withdrawing from noxious or innocuous stimuli)	Pain	C/E	Evaluate whether grasp or withdrawal was appropriate given noxious or innocuous stimulus	Pain > no pain
Nomi et al., 2008	1.5T	14 (7F)	10	Facial expressions (photos)	Happy, sad, neutral	C/E	Instructed to recognize and share emotion	Emotional > control
Nummenmaa et al., 2008		10 (10F)	8	Aversive threatening scenes (photos)	Negative	C/E	Empathize with aggressor or victim	Emotional > neutral
Osborn et al., 2010	3T	Resp: 10 (6F); non-resp 10 (6F)	6	Scenes (faces plus context)	Pain	A/P		Pain > control (resp)<br>Pain > control (non-resp)<br>*Control > pain (non-resp)*

Paulus et al., 2017	3T	17 (0F)	8	Drawings	Empathic embarrassment	C/E	Rate how much vicarious embarrassment they felt	Empathic embarrassment > neutral
Pehrs et al., 2017	3T	26 (17F)	6	Empathic film with sad music	Sadness	C/E	Rate 'moving' feeling and level of compassion	Sad > neutral
Powell et al., 2017	3T	12 (0F)	6	Drawings (comic strip)	Does not specify	C/E	Asked to choose the right ending to the comic	Empathic inference > control (physical causality inference)
Preis et al., 2013	3T	64 (32F)	8	Pain infliction to body part (photo)	Pain	C/E	Imagine how the person in photo feels	Pain > no pain<br>*No pain > pain*
Preston et al., 2007	PET	16 (8F)	16	Emotional scenarios (imagining)	Fear or anger	C/E	Imagine emotional scenarios	Emotional > neutral (personal)<br>Emotional > neutral (non-personal)
Prochnow et al., 2013	3T	15 (7F)	8	Facial expressions (video clips)	Anger, sadness, fear, disgust, surprise	C/E	Rate the emotion in the video clip	Emotional > control
Prochnow et al., 2014	3T	26 (12F)	6	Facial expressions (photos)	Happiness, anger, sadness, fear	C/E	Match descriptive sentences to facial expressions	Emotional > control
Regenbogen et al., 2012	3T	27 (13F)	8	Facial expressions and descriptions (video clips)	Disgust, fear, happy, sad	A/P		Emotional vs. neutral
Reniers et al., 2014	3T	15 (0F)	7x7x10	Emotional scenes	Sad vs. neutral	C/E	Imagine what another is feeling	Sad > neutral
Riekki et al., 2017	3T	38 (19F)	8	Facial expressions (photos)	Pain, injury, grief	A/P<br>C/E	n/a<br>Rate how emotionally touching the photo is	Emotional > neutral (passive)<br>Emotional > neutral (active)
Riem et al., 2011	3T	42 (42F)	5	Crying (auditory)	Distress	A/P		Distress > neutral
Ruckmann et al., 2015	3T	30 (15F)	8	Pain infliction to body part (photo)	Pain	C/E	Rate the pain level of people in photo	Pain > no pain
Schneider et al., 2013	3T	28 (13F)	8	Emotional videos	Disgust, happiness, fear, anger	C/E	Asked about what they felt and what other felt	Emotional > neutral
Schulte-Ruther et al., 2008	1.5T	26 (14F)	NR	Facial expressions (photos)	Fear, anger	C/E	Self-evaluated how they felt	Fear/Anger > Neutral (self)<br>Fear/Anger > Neutral (other)

							Other-evaluated how the person in the picture felt	
Seara-Cardoso et al., 2015	1.5T	46 (0F)	8	Pain infliction to body part (photo)	Pain	A/P		Pain > no pain
Seara-Cardoso et al., 2016	1.5T	31 (0F)	8	Facial expressions (photos)	Sad, fearful, angry, happy	A/P		Emotional > neutral
Sebastian et al., 2012	1.5T	30 (0F)	8	Drawings (comic strips)	Not specified	C/E	Choose proper ending to comic strip	Emotional inference > control (physical causality inference)
Sheng et al., 2014	3T	21 (11F)	8	Facial expressions (photos)	Pain	C/E	Judge whether or not the other person is in pain	Pain > no pain
Simon et al., 2006	1.5T	17 (9F)	10	Facial expressions (photos)	Pain, anger	A/P		Pain > no pain<br>Angry > neutral
Simon-Thomas et al., 2012	4T	17 (11F)	8	Emotional scenes (photos)	Compassion/ distress	C/E	Rate their emotional response to scenes	Distress > neutral
Singer et al., 2004	1.5T	16 (16F)	10	Real life (heat probe on hand in mirror)	Pain	A/P		Pain > no pain
Singh et al., 2015	3T	14 (4F)	6	Emotional scenes (photos)	Suffering/distress	C/E	Rate their emotional response to scenes	Suffering > neutral
Spencer et al., 2011	3T	40 (20F)	10	Facial expressions (photos)	Fear, happiness	A/P		Happy > neutral<br>Fear > neutral
Szicik et al., 2017	3T	30 (0F)	8	Drawings	Distress	C/E	Imagine how they would feel in situation	Distress > neutral
Toller et al., 2015	3T	30 (17F)	4	Facial expressions (video clips)	Fear	A/P		Fear > control
Tusche et al., 2016	3T	31 (15F)	8	Facial expressions and descriptions (video clips)	Negative	C/E	Rate their own affect and compassion	Emotionally negative > neutral
Ushida et al., 2008	1.5T	15 (8F)	8	Pain infliction to body part (photos)	Pain/fear	A/P		Pain > no pain
Vachon-Presseau et al., 2012	3T	20 (10F)	6	Pain infliction to body part (photo)	Pain	C/E	Evaluate whether person is in pain or not	Pain > no pain
Van der Heiden et al., 2013	1.5T	18 (9F)	8	Pain infliction to body part (photo)	Pain	A/P		Pain > no pain
Vollm et al., 2006	1.5T	13 (0F)	10	Drawings (comic strip)	Emotion - doesn't specifically say	C/E	Imagine how main character feels	Empathic inference > control (physical causality inference)

Wang et al., 2015	3T	56 (25F)	10	Drawings (comic strip)	Not specified	C/E	Infer proper ending to the comic strip	Empathic inference > control (physical causality inference)
Warren et al., 2006	3T	20 (12F)	8	Nonverbal vocal expressions (auditory)	Positive (triumph), negative (fear, disgust)	A/P		Emotion > baseline
Wicker et al., 2003	3T	14 (0F)	6	Facial expressions (video clips) plus scents	Disgust, pleasure	Not specif ied		Disgust > neutral<br>Pleasure > neutral
Xu et al., 2009	3T	33 (17F)	8	Pain infliction to body part (animated)	Pain	C/E	Evaluate whether person is in pain or not	Pain > no pain

AIR = Automated Image Registration; NR = Not Reported; T = Tesla (field strength MRI scanner)

BIBLIOGRAPHY

Akitsuki, Y., and Decety, J. (2009). Social context and perceived agency affects empathy for pain: An event-related fMRI investigation. *NeuroImage* 47**,** 722-734.

Arbuckle, N.L., and Shane, M.S. (2017). Up-regulation of neural indicators of empathic concern in an offender population. *Soc Neurosci* 12**,** 386-390.

Azevedo, R.T., Macaluso, E., Avenanti, A., Santangelo, V., Cazzato, V., and Aglioti, S.M. (2013). Their pain is not our pain: brain and autonomic correlates of empathic resonance with the pain of same and different race individuals. *Hum Brain Mapp* 34**,** 3168-3181.

Azevedo, R.T., Macaluso, E., Viola, V., Sani, G., and Aglioti, S.M. (2014). Weighing the stigma of weight: An fMRI study of neural reactivity to the pain of obese individuals. *NeuroImage* 91**,** 109-119.

Aziz-Zadeh, L., Sheng, T., Liew, S.L., and Damasio, H. (2012). Understanding otherness: The neural bases of action comprehension and pain empathy in a congenital amputee. *Cerebral Cortex* 22**,** 811-819.

Bagozzi, R.P., Verbeke, W.J.M.I., Dietvorst, R.C., Belschak, F.D., Van Den Berg, W.E., and Rietdijk, W.J.R. (2013). Theory of mind and empathic explanations of Machiavellianism: A neuroscience perspective. *Journal of Management* 39**,** 1760-1798.

Benuzzi, F., Lui, F., Duzzi, D., Nichelli, P.F., and Porro, C.A. (2008). Does it look painful or disgusting? Ask your parietal and cingulate cortex. *Journal of Neuroscience* 28**,** 923-931.

Berlingeri, M., Gallucci, M., Danelli, L., Forgiarini, M., Sberna, M., and Paulesu, E. (2016). Guess who's coming to dinner: Brain signatures of racially biased and politically correct behaviors. *Neuroscience* 332**,** 231-241.

Bodden, M.E., Kubler, D., Knake, S., Menzler, K., Heverhagen, J.T., Sommer, J., Kalbe, E., Krach, S., and Dodel, R. (2013). Comparing the neural correlates of affective and cognitive theory of mind using fMRI: Involvement of the basal ganglia in affective theory of mind. *Adv Cogn Psychol* 9**,** 32-43.

Bos, P.A., Montoya, E.R., Hermans, E.J., Keysers, C., and Van Honk, J. (2015). Oxytocin reduces neural activity in the pain circuitry when seeing pain in others. *NeuroImage* 113**,** 217-224.

Botvinick, M., Jha, A.P., Bylsma, L.M., Fabian, S.A., Solomon, P.E., and Prkachin, K.M. (2005). Viewing facial expressions of pain engages cortical areas involved in the direct experience of pain. *Neuroimage* 25**,** 312-319.

Bruneau, E.G., Dufour, N., and Saxe, R. (2012a). Social cognition in members of conflict groups: Behavioural and neural responses in Arabs, Israelis and South Americans to each other's misfortunes. *Philosophical Transactions of the Royal Society B: Biological Sciences* 367**,** 717-730.

Bruneau, E.G., Pluta, A., and Saxe, R. (2012b). Distinct roles of the 'Shared Pain' and 'Theory of Mind' networks in processing others' emotional suffering. *Neuropsychologia* 50**,** 219-231.

Brunnlieb, C., Münte, T., Tempelmann, C., and Heldmann, M. 2013. Vasopressin modulates neural responses related to emotional stimuli in the right amygdala. *Brain research* [Online], 1499. Available: http://onlinelibrary.wiley.com/o/cochrane/clcentral/articles/997/CN-00965997/frame.html.

Budell, L., Jackson, P., and Rainville, P. (2010). Brain responses to facial expressions of pain: Emotional or motor mirroring? *NeuroImage* 53**,** 355-363.

Cheng, Y., Chen, C., Lin, C.P., Chou, K.H., and Decety, J. (2010). Love hurts: An fMRI study. *NeuroImage* 51**,** 923-929.

Cheng, Y., Lin, C.P., Liu, H.L., Hsu, Y.Y., Lim, K.E., Hung, D., and Decety, J. (2007). Expertise Modulates the Perception of Pain in Others. *Current Biology* 17**,** 1708-1713.

Cheon, B.K., Im, D.M., Harada, T., Kim, J.S., Mathur, V.A., Scimeca, J.M., Parrish, T.B., Park, H., and Chiao, J.Y. (2013). Cultural modulation of the neural correlates of emotional pain perception: The role of other-focusedness. *Neuropsychologia* 51**,** 1177-1186.

Chiao, J.Y., Mathur, V.A., Harada, T., and Lipke, T. (2009). "Neural basis of preference for human social hierarchy versus egalitarianism".).

Choe, D.E., Shaw, D.S., and Forbes, E.E. (2015). Maladaptive social information processing in childhood predicts young men's atypical amygdala reactivity to threat. *Journal of child psychology and psychiatry, and allied disciplines* 56**,** 549-557.

Coll, M.P., Grégoire, M., Eugène, F., and Jackson, P.L. (2017). Neural correlates of prosocial behavior towards persons in pain in healthcare providers. *Biological Psychology* 128**,** 1-10.

Corradi-Dell'acqua, C., Hofstetter, C., and Vuilleumier, P. (2011). Felt and seen pain evoke the same local patterns of cortical activity in insular and cingulate cortex. *Journal of Neuroscience* 31**,** 17996-18006.

Danziger, N., Faillenot, I., and Peyron, R. (2009). Can we share a pain we never felt? Neural correlates of empathy in patients with congenital insensitivity to pain. *European Journal of Pain* 13**,** S33.

Dapretto, M., Davies, M.S., Pfeifer, J.H., Scott, A.A., Sigman, M., Bookheimer, S.Y., and Iacoboni, M. (2006). Understanding emotions in others: mirror neuron dysfunction in children with autism spectrum disorders. *Nat Neurosci* 9**,** 28-30.

De Gelder, B., Snyder, J., Greve, D., Gerard, G., and Hadjikhani, N. (2004). Fear fosters flight: A mechanism for fear contagion when perceiving emotion expressed by a whole body. *PNAS* 101**,** 16701-16706.

De Greck, M., Scheidt, L., Bolter, A.F., Frommer, J., Ulrich, C., Stockum, E., Enzi, B., Tempelmann, C., Hoffmann, T., Han, S., and Northoff, G. (2012a). Altered brain activity during emotional empathy in somatoform disorder. *Hum Brain Mapp* 33**,** 2666-2685.

De Greck, M., Wang, G., Yang, X., Wang, X., Northoff, G., and Han, S. (2012b). Neural substrates underlying intentional empathy. *Social cognitive and affective neuroscience* 7**,** 135-144.
Decety, J., and Michalska, K.J. (2010). Neurodevelopmental changes in the circuits underlying empathy and sympathy from childhood to adulthood. *Developmental Science* 13**,** 886-899.
Decety, J., Michalska, K.J., and Akitsuki, Y. (2008). Who caused the pain? An fMRI investigation of empathy and intentionality in children. *Neuropsychologia* 46**,** 2607-2614.
Decety, J., Michalska, K.J., Akitsuki, Y., and Lahey, B.B. (2009). Atypical empathic responses in adolescents with aggressive conduct disorder: A functional MRI investigation. *Biological Psychology* 80**,** 203-211.
Deeley, Q., Daly, E., Surguladze, S., Tunstall, N., Mezey, G., Beer, D., Ambikapathy, A., Robertson, D., Giampietro, V., Brammer, M.J., Clarke, A., Dowsett, J., Fahy, T., Phillips, M.L., and Murphy, D.G. (2006). Facial emotion processing in criminal psychopathy: Preliminary functional magnetic resonance imaging study. *British Journal of Psychiatry* 189**,** 533-539.
Dong, D., Ming, Q., Wang, X., Yu, W., Jiang, Y., Wu, Q., Gao, Y., and Yao, S. (2016). Temporoparietal Junction Hypoactivity during Pain-Related Empathy Processing in Adolescents with Conduct Disorder. *Front Psychol* 7**,** 2085.
Enzi, B., Amirie, S., and Brune, M. (2016). Empathy for pain-related dorsolateral prefrontal activity is modulated by angry face perception. *Exp Brain Res* 234**,** 3335-3345.
Ernst, J., Northoff, G., Boker, H., Seifritz, E., and Grimm, S. (2013). Interoceptive awareness enhances neural activity during empathy. *Hum Brain Mapp* 34**,** 1615-1624.
Fan, Y.T., Chen, C., Chen, S.C., Decety, J., and Cheng, Y. (2014). Empathic arousal and social understanding in individuals with autism: evidence from fMRI and ERP measurements. *Social cognitive and affective neuroscience* 9**,** 1203-1213.
Feng, C., Li, Z., Feng, X., Wang, L., Tian, T., and Luo, Y.J. (2016). Social hierarchy modulates neural responses of empathy for pain. *Social cognitive and affective neuroscience* 11**,** 485-495.
Fourie, M.M., Stein, D.J., Solms, M., Gobodo-Madikizela, P., and Decety, J. (2017). Empathy and moral emotions in post-apartheid South Africa: an fMRI investigation. *Soc Cogn Affect Neurosci* 12**,** 881-892.
Fujino, J., Yamasaki, N., Miyata, J., Kawada, R., Sasaki, H., Matsukawa, N., Takemura, A., Ono, M., Tei, S., Takahashi, H., Aso, T., Fukuyama, H., and Murai, T. (2014). Altered brain response to others' pain in major depressive disorder. *Journal of Affective Disorders* 165**,** 170-175.
Gao, X., Pan, W., Li, C., Weng, L., Yao, M., and Chen, A. (2017). Long-Time Exposure to Violent Video Games Does Not Show Desensitization on Empathy for Pain: An fMRI Study. *Front Psychol* 8**,** 650.
Geday, J., Gjedde, A., Boldsen, A.S., and Kupers, R. (2003). Emotional valence modulates activity in the posterior fusiform gyrus and inferior medial prefrontal cortex in social perception. *NeuroImage* 18**,** 675-684.
Geday, J., Kupers, R., and Gjedde, A. (2007). As time goes by: temporal constraints on emotional activation of inferior medial prefrontal cortex. *Cereb Cortex* 17**,** 2753-2759.
Göttlich, M., Ye, Z., Rodriguez-Fornells, A., Münte, T.F., and Krämer, U.M. (2017). Viewing socio-affective stimuli increases connectivity within an extended default mode network. *NeuroImage* 148**,** 8-19.

Greimel, E., Schulte-Ruther, M., Fink, G.R., Piefke, M., Herpertz-Dahlmann, B., and Konrad, K. (2010). Development of neural correlates of empathy from childhood to early adulthood: an fMRI study in boys and adult men. *J Neural Transm (Vienna)* 117**,** 781-791.

Grice-Jackson, T., Critchley, H.D., Banissy, M.J., and Ward, J. (2017). Consciously feeling the pain of others reflects atypical functional connectivity between the pain matrix and frontal-parietal regions. *Frontiers in Human Neuroscience* 11.

Grosbras, M.H., and Paus, T. (2006). Brain networks involved in viewing angry hands or faces. *Cerebral Cortex* 16**,** 1087-1096.

Gu, X., Liu, X., Guise, K.G., Naidich, T.P., Hof, P.R., and Fan, J. (2010). Functional dissociation of the frontoinsular and anterior cingulate cortices in empathy for pain. *Journal of Neuroscience* 30**,** 3739-3744.

Gu, X., Liu, X., Van Dam, N.T., Hof, P.R., and Fan, J. (2013). Cognition-emotion integration in the anterior insular cortex. *Cerebral Cortex* 23**,** 20-27.

Hadjikhani, N., Zurcher, N.R., Rogier, O., Hippolyte, L., Lemonnier, E., Ruest, T., Ward, N., Lassalle, A., Gillberg, N., Billstedt, E., Helles, A., Gillberg, C., Solomon, P., Prkachin, K.M., and Gillberg, C. (2014). Emotional contagion for pain is intact in autism spectrum disorders. *Transl Psychiatry* 4**,** e343.

Han, S., Fan, Y., Xu, X., Qin, J., Wu, B., Wang, X., Aglioti, S.M., and Mao, L. (2009). Empathic neural responses to others' pain are modulated by emotional contexts. *Human Brain Mapping* 30**,** 3227-3237.

Han, X., He, K., Wu, B., Shi, Z., Liu, Y., Luo, S., Wei, K., Wu, X., and Han, S. (2017). Empathy for pain motivates actions without altruistic effects: evidence of motor dynamics and brain activity. *Soc Cogn Affect Neurosci* 12**,** 893-901.

Horan, W.P., Iacoboni, M., Cross, K.A., Korb, A., Lee, J., Nori, P., Quintana, J., Wynn, J.K., and Green, M.F. (2014). Self-reported empathy and neural activity during action imitation and observation in schizophrenia. *NeuroImage. Clinical* 5**,** 100-108.

Jackson, P.L., Latimer, M., Eugene, F., Macleod, E., Hatfield, T., Vachon-Presseau, E., Michon, P.E., and Prkachin, K.M. (2017). Empathy in paediatric intensive care nurses part 2: Neural correlates. *J Adv Nurs* 73**,** 2686-2695.

Jankowiak-Siuda, K., Rymarczyk, K., Zurawski, L., Jednorog, K., and Marchewka, A. (2015). Physical attractiveness and sex as modulatory factors of empathic brain responses to pain. *Front Behav Neurosci* 9**,** 236.

Jensen, K.B., Petrovic, P., Kerr, C.E., Kirsch, I., Raicek, J., Cheetham, A., Spaeth, R., Cook, A., Gollub, R.L., Kong, J., and Kaptchuk, T.J. (2014). *Sharing pain and relief: Neural correlates of physicians during treatment of patients.*

Kana, R.K., Patriquin, M.A., Black, B.S., Channell, M.M., and Wicker, B. (2016). Altered Medial Frontal and Superior Temporal Response to Implicit Processing of Emotions in Autism. *Autism Research* 9**,** 55-66.

Kanske, P., Böckler, A., Trautwein, F.M., and Singer, T. (2015). Dissecting the social brain: Introducing the EmpaToM to reveal distinct neural networks and brain-behavior relations for empathy and Theory of Mind. *NeuroImage* 122**,** 6-19.

Kim, E., Jung, Y.C., Ku, J., Kim, J.J., Lee, H., Kim, S.Y., Kim, S.I., and Cho, H.S. (2009a). Reduced activation in the mirror neuron system during a virtual social cognition task in euthymic bipolar disorder. *Progress in Neuro-Psychopharmacology and Biological Psychiatry* 33**,** 1409-1416.

Kim, J.-W., Kim, S.-E., Kim, J.-J., Jeong, B., Park, C.-H., Son, A.R., Song, J.E., and Ki, S.W. (2009b). Compassionate attitude towards others' suffering activates the mesolimbic neural system. *Neuropsychologia* 47**,** 2073-2081.

Kim, Y.-T., Lee, J.-J., Song, H.-J., Kim, J.-H., Kwon, D.-H., Kim, M.-N., Yoo, D.-S., Lee, H.J., Kim, H.-J., and Chang, Y. (2010). Alterations in cortical activity of male methamphetamine abusers performing an empathy task: fMRI study. *Human Psychopharmacology: Clinical and Experimental* 25**,** 63-70.

Kim, Y.T., Song, H.J., Seo, J.H., Lee, J.J., Lee, J., Kwon, D.H., Yoo, D.S., Lee, H.J., Suh, K.J., and Chang, Y. (2011). The differences in neural network activity between methamphetamine abusers and healthy subjects performing an emotion-matching task: Functional MRI study. *NMR in Biomedicine* 24**,** 1392-1400.

Klimecki, O.M., Leiberg, S., Lamm, C., and Singer, T. (2013). Functional neural plasticity and associated changes in positive affect after compassion training. *Cerebral Cortex* 23**,** 1552-1561.

Krach, S., Kamp-Becker, I., Einhäuser, W., Sommer, J., Frässle, S., Jansen, A., Rademacher, L., Müller-Pinzler, L., Gazzola, V., and Paulus, F.M. (2015). Evidence from pupillometry and fMRI indicates reduced neural response during vicarious social pain but not physical pain in autism. *Human Brain Mapping* 36**,** 4730-4744.

Kramer, U.M., Mohammadi, B., Donamayor, N., Samii, A., and Munte, T.F. (2010). Emotional and cognitive aspects of empathy and their relation to social cognition-An fMRI-study. *Brain Research* 1311**,** 110-120.

Labek, K., Berger, S., Buchheim, A., Bosch, J., Spohrs, J., Dommes, L., Beschoner, P., Stingl, J.C., and Viviani, R. (2017). The iconography of mourning and its neural correlates: a functional neuroimaging study. *Soc Cogn Affect Neurosci* 12**,** 1303-1313.

Lamm, C., and Decety, J. (2008). Is the extrastriate body area (EBA) sensitive to the perception of pain in others? *Cerebral Cortex* 18**,** 2369-2373.

Lang, S., Yu, T., Markl, A., Müller, F., and Kotchoubey, B. (2011). Hearing others' pain: Neural activity related to empathy. *Cognitive, Affective and Behavioral Neuroscience* 11**,** 386-395.

Lee, K.H., Brown, W.H., Egleston, P.N., Green, R.D., Farrow, T.F., Hunter, M.D., Parks, R.W., Wilkinson, I.D., Spence, S.A., and Woodruff, P.W. (2006). A functional magnetic resonance imaging study of social cognition in schizophrenia during an acute episode and after recovery. *Am J Psychiatry* 163**,** 1926-1933.

Lee, S.J., Kang, D.H., Kim, C.-W., Gu, B.M., Park, J.-Y., Choi, C.-H., Shin, N.Y., Lee, J.-M., and Kwon, J.S. (2010). Multi-level comparison of empathy in schizophrenia: An fMRI study of a cartoon task. *Psychiatry Research: Neuroimaging* 181**,** 121-129.

Lee, T.M.C., Sun, D., Leung, M.K., Chu, L.W., and Keysers, C. (2013). Neural activities during affective processing in people with Alzheimer's disease. *Neurobiology of Aging* 34**,** 706-715.

Leiberg, S., Eippert, F., Veit, R., and Anders, S. (2012). Intentional social distance regulation alters affective responses towards victims of violence: An FMRI study. *Human Brain Mapping* 33**,** 2464-2476.

Lenzi, D., Trentini, C., Pantano, P., Macaluso, E., Lenzi, G.L., and Ammaniti, M. (2013). Attachment models affect brain responses in areas related to emotions and empathy in nulliparous women. *Human Brain Mapping* 34**,** 1399-1414.

Luo, S., Li, B., Ma, Y., Zhang, W., Rao, Y., and Han, S. (2015). Oxytocin receptor gene and racial ingroup bias in empathy-related brain activity. *NeuroImage* 110**,** 22-31.

Luo, S., Shi, Z., Yang, X., Wang, X., and Han, S. (2014). Reminders of mortality decrease midcingulate activity in response to others' suffering. *Social cognitive and affective neuroscience* 9**,** 477-486.

Mascaro, J.S., Hackett, P.D., Gouzoules, H., Lori, A., and Rilling, J.K. (2014). Behavioral and genetic correlates of the neural response to infant crying among human fathers. *Social cognitive and affective neuroscience* 9**,** 1704-1712.

Mathur, V.A., Cheon, B.K., Harada, T., Scimeca, J.M., and Chiao, J.Y. (2016). Overlapping neural response to the pain or harm of people, animals, and nature. *Neuropsychologia* 81**,** 265-273.

Mathur, V.A., Harada, T., Lipke, T., and Chiao, J.Y. (2010). Neural basis of extraordinary empathy and altruistic motivation. *NeuroImage* 51**,** 1468-1475.

Mazza, M., Tempesta, D., Pino, M.C., Catalucci, A., Gallucci, M., and Ferrara, M. (2013). Regional cerebral changes and functional connectivity during the observation of negative emotional stimuli in subjects with post-traumatic stress disorder. *European Archives of Psychiatry and Clinical Neuroscience* 263**,** 575-583.

Mazzola, V., Latorre, V., Petito, A., Gentili, N., Fazio, L., Popolizio, T., Blasi, G., Arciero, G., and Bondolfi, G. (2010). Affective response to a loved one's pain: Insula activity as a function of individual differences. *PLoS ONE* 5.

Melchers, M., Markett, S., Montag, C., Trautner, P., Weber, B., Lachmann, B., Buss, P., Heinen, R., and Reuter, M. (2015). Reality TV and vicarious embarrassment: An fMRI study. *NeuroImage* 109**,** 109-117.

Mercadillo, R.E., Alcauter, S., Fernandez-Ruiz, J., and Barrios, F.A. (2015). Police culture influences the brain function underlying compassion: a gender study. *Soc Neurosci* 10**,** 135-152.

Mercadillo, R.E., Díaz, J.L., Pasaye, E.H., and Barrios, F.A. (2011). Perception of suffering and compassion experience: Brain gender disparities. *Brain and Cognition* 76**,** 5-14.

Michalska, K.J., Kinzler, K.D., and Decety, J. (2013). Age-related sex differences in explicit measures of empathy do not predict brain responses across childhood and adolescence. *Developmental Cognitive Neuroscience* 3**,** 22-32.

Molenberghs, P., Gapp, J., Wang, B., Louis, W.R., and Decety, J. (2016). Increased Moral Sensitivity for Outgroup Perpetrators Harming Ingroup Members. *Cereb Cortex* 26**,** 225-233.

Moll, J., De Oliveira-Souza, R., Garrido, G.J., Bramati, I.E., Caparelli-Daquer, E.M.A., Paiva, M.L.M.F., Zahn, R., and Grafman, J. (2007). The self as a moral agent: Linking the neural bases of social agency and moral sensitivity. *Social Neuroscience* 2**,** 336-352.

Montoya, J.L., Landi, N., Kober, H., Worhunsky, P.D., Rutherford, H.J.V., Mencl, W.E., Mayes, L.C., and Potenza, M.N. (2012). Regional brain responses in nulliparous women to emotional infant stimuli. *PLoS ONE* 7.

Morelli, S.A., Rameson, L.T., and Lieberman, M.D. (2014). The neural components of empathy: predicting daily prosocial behavior. *Social cognitive and affective neuroscience* 9**,** 39-47.

Moriguchi, Y., Decety, J., Ohnishi, T., Maeda, M., Mori, T., Nemoto, K., Matsuda, H., and Komaki, G. (2007). Empathy and judging other's pain: An fMRI study of alexithymia. *Cerebral Cortex* 17**,** 2223-2234.

Morrison, I., and Downing, P.E. (2007). Organization of felt and seen pain responses in anterior cingulate cortex. *Neuroimage* 37**,** 642-651.

Morrison, I., Lloyd, D., Di Pellegrino, G., and Roberts, N. (2004). Vicarious responses to pain in anterior cingulate cortex: Is empathy a multisensory issue? *Cognitive, Affective and Behavioral Neuroscience* 4**,** 270-278.

Morrison, I., Peelen, M.V., and Downing, P.E. (2007). The sight of others' pain modulates motor processing in human cingulate cortex. *Cerebral Cortex* 17**,** 2214-2222.

Morrison, I., Tipper, S.P., Fenton-Adams, W.L., and Bach, P. (2013). "Feeling" others' painful actions: The sensorimotor integration of pain and action information. *Human Brain Mapping* 34**,** 1982-1998.

Nomi, J.S., Scherfeld, D., Friederichs, S., Schafer, R., Franz, M., Wittsack, H.-J., Azari, N.P., Missimer, J., and Seitz, R.J. (2008). On the neural networks of empathy: A principal component analysis of an fMRI study. *Behavioral and Brain Functions* 4.

Nummenmaa, L., Hirvonen, J., Parkkola, R., and Hietanen, J.K. (2008). Is emotional contagion special? An fMRI study on neural systems for affective and cognitive empathy. *NeuroImage* 43**,** 571-580.

Osborn, J., and Derbyshire, S.W.G. (2010). Pain sensation evoked by observing injury in others. *Pain* 148**,** 268-274.

Paulus, F.M., Müller-Pinzler, L., Stolz, D.S., Mayer, A.V., Rademacher, L., and Krach, S. (2017). Laugh or cringe? Common and distinct processes of reward-based schadenfreude and empathy-based fremdscham. *Neuropsychologia*.

Pehrs, C., Zaki, J., Schlochtermeier, L.H., Jacobs, A.M., Kuchinke, L., and Koelsch, S. (2017). The Temporal Pole Top-Down Modulates the Ventral Visual Stream During Social Cognition. *Cereb Cortex* 27**,** 777-792.

Powell, J.L., Grossi, D., Corcoran, R., Gobet, F., and García-Fiñana, M. (2017). The neural correlates of theory of mind and their role during empathy and the game of chess: A functional magnetic resonance imaging study. *Neuroscience* 355**,** 149-160.

Preis, M.A., Schmidt-Samoa, C., Dechent, P., and Kroener-Herwig, B. (2013). The effects of prior pain experience on neural correlates of empathy for pain: An fMRI study. *Pain* 154**,** 411-418.

Preston, S.D., Bechara, A., Damasio, H., Grabowski, T.J., Stansfield, R.B., Mehta, S., and Damasio, A.R. (2007). The neural substrates of cognitive empathy. *Social Neuroscience* 2**,** 254-275.

Prochnow, D., Brunheim, S., Steinhäuser, L., and Seitz, R.J. (2014). Reasoning about the implications of facial expressions: A behavioral and fMRI study on low and high social impact. *Brain and Cognition* 90**,** 165-173.

Prochnow, D., Höing, B., Kleiser, R., Lindenberg, R., Wittsack, H.J., Schäfer, R., Franz, M., and Seitz, R.J. (2013). The neural correlates of affect reading: An fMRI study on faces and gestures. *Behavioural Brain Research* 237**,** 270-277.

Regenbogen, C., Schneider, D.A., Gur, R.E., Schneider, F., Habel, U., and Kellermann, T. (2012). Multimodal human communication - Targeting facial expressions, speech content and prosody. *NeuroImage* 60**,** 2346-2356.

Reniers, R.L., Vollm, B.A., Elliott, R., and Corcoran, R. (2014). Empathy, ToM, and self-other differentiation: an fMRI study of internal states. *Soc Neurosci* 9**,** 50-62.

Riekki, T., Svedholm-Hakkinen, A.M., and Lindeman, M. (2017). Empathizers and systemizers process social information differently. *Soc Neurosci***,** 1-12.

Riem, M.M.E., Bakermans-Kranenburg, M.J., Pieper, S., Tops, M., Boksem, M.a.S., Vermeiren, R.R.J.M., Van Ijzendoorn, M.H., and Rombouts, S.a.R.B. (2011). Oxytocin modulates amygdala, insula, and inferior frontal gyrus responses to infant crying: A randomized controlled trial. *Biological Psychiatry* 70**,** 291-297.

Ruckmann, J., Bodden, M., Jansen, A., Kircher, T., Dodel, R., and Rief, W. (2015). How pain empathy depends on ingroup/outgroup decisions: A functional magnet resonance imaging study. *Psychiatry Research - Neuroimaging* 234**,** 57-65.

Schneider, K., Pauly, K.D., Gossen, A., Mevissen, L., Michel, T.M., Gur, R.C., Schneider, F., and Habel, U. (2013). Neural correlates of moral reasoning in autism spectrum disorder. *Social cognitive and affective neuroscience* 8**,** 702-710.

Schulte-Rüther, M., Markowitsch, H.J., Shah, N.J., Fink, G.R., and Piefke, M. (2008). Gender differences in brain networks supporting empathy. *NeuroImage* 42**,** 393-403.

Seara-Cardoso, A., Sebastian, C.L., Viding, E., and Roiser, J.P. (2016). Affective resonance in response to others' emotional faces varies with affective ratings and psychopathic traits in amygdala and anterior insula. *Soc Neurosci* 11**,** 140-152.

Seara-Cardoso, A., Viding, E., Lickley, R.A., and Sebastian, C.L. (2015). Neural responses to others' pain vary with psychopathic traits in healthy adult males. *Cognitive, affective & behavioral neuroscience* 15**,** 578-588.

Sebastian, C.L., Fontaine, N.M., Bird, G., Blakemore, S.J., Brito, S.A., Mccrory, E.J., and Viding, E. (2012). Neural processing associated with cognitive and affective Theory of Mind in adolescents and adults. *Social cognitive and affective neuroscience* 7**,** 53-63.

Sheng, F., Liu, Q., Li, H., Fang, F., and Han, S. (2014). Task modulations of racial bias in neural responses to others' suffering. *NeuroImage* 88**,** 263-270.

Simon-Thomas, E.R., Godzik, J., Castle, E., Antonenko, O., Ponz, A., Kogan, A., and Keltner, D.J. (2012). An fMRI study of caring vs self-focus during induced compassion and pride. *Social cognitive and affective neuroscience* 7**,** 635-648.

Simon, D., Craig, K.D., Miltner, W.H., and Rainville, P. (2006). Brain responses to dynamic facial expressions of pain. *Pain* 126**,** 309-318.

Singer, T., Seymour, B., O'doherty, J., Kaube, H., Dolan, R.J., and Frith, C.D. (2004). Empathy for Pain Involves the Affective but not Sensory Components of Pain. *Science* 303**,** 1157-1162.

Singh, S., Modi, S., Goyal, S., Kaur, P., Singh, N., Bhatia, T., Deshpande, S.N., and Khushu, S. (2015). Functional and structural abnormalities associated with empathy in patients with schizophrenia: An fMRI and VBM study. *Journal of Biosciences* 40**,** 355-364.

Spencer, M.D., Holt, R.J., Chura, L.R., Suckling, J., Calder, A.J., Bullmore, E.T., and Baron-Cohen, S. (2011). A novel functional brain imaging endophenotype of autism: the neural response to facial expression of emotion. *Translational psychiatry* 1**,** e19.

Szycik, G.R., Mohammadi, B., Munte, T.F., and Te Wildt, B.T. (2017). Lack of Evidence That Neural Empathic Responses Are Blunted in Excessive Users of Violent Video Games: An fMRI Study. *Front Psychol* 8**,** 174.

Toller, G., Adhimoolam, B., Grunwald, T., Huppertz, H.J., Kurthen, M., Rankin, K.P., and Jokeit, H. (2015). Right mesial temporal lobe epilepsy impairs empathy-related brain responses to dynamic fearful faces. *Journal of Neurology* 262**,** 729-741.

Tusche, A., Bockler, A., Kanske, P., Trautwein, F.M., and Singer, T. (2016). Decoding the Charitable Brain: Empathy, Perspective Taking, and Attention Shifts Differentially Predict Altruistic Giving. *J Neurosci* 36**,** 4719-4732.

Ushida, T., Ikemoto, T., Tanaka, S., Shinozaki, J., Taniguchi, S., Murata, Y., Mclaughlin, M., Arai, Y.C., and Tamura, Y. (2008). Virtual needle pain stimuli activates cortical representation of emotions in normal volunteers. *Neurosci Lett* 439**,** 7-12.

Vachon-Presseau, E., Roy, M., Martel, M.O., Albouy, G., Chen, J., Budell, L., Sullivan, M.J., Jackson, P.L., and Rainville, P. (2012). Neural processing of sensory and emotional-communicative information associated with the perception of vicarious pain. *NeuroImage* 63**,** 54-62.

Van Der Heiden, L., Scherpiet, S., Konicar, L., Birbaumer, N., and Veit, R. (2013). Inter-individual differences in successful perspective taking during pain perception mediates emotional responsiveness in self and others: An fMRI study. *NeuroImage* 65**,** 387-394.

Vollm, B.A., Taylor, A.N., Richardson, P., Corcoran, R., Stirling, J., Mckie, S., Deakin, J.F., and Elliott, R. (2006). Neuronal correlates of theory of mind and empathy: a functional magnetic resonance imaging study in a nonverbal task. *Neuroimage* 29**,** 90-98.

Wang, Y., Liu, W.H., Li, Z., Wei, X.H., Jiang, X.Q., Neumann, D.L., Shum, D.H., Cheung, E.F., and Chan, R.C. (2015). Dimensional schizotypy and social cognition: an fMRI imaging study. *Front Behav Neurosci* 9**,** 133.

Warren, J.E., Sauter, D.A., Eisner, F., Wiland, J., Dresner, M.A., Wise, R.J., and Scott, S.K. (2006). Positive emotions preferentially engage an auditory-motor "mirror" system. *Journal of Neuroscience* 26**,** 13067-13075.

Wicker, B., Keysers, C., Plailly, J., Royet, J.P., Gallese, V., and Rizzolatti, G. (2003). Both of us disgusted in My insula: the common neural basis of seeing and feeling disgust *Neuron* 40**,** 655-664.

Xu, X., Zuo, X., Wang, X., and Han, S. (2009). Do you feel my pain? Racial group membership modulates empathic neural responses. *Journal of Neuroscience* 29**,** 8525-8529.
