# Supplementary material for: Is Empathy for Pain Unique in Its Neural Correlates? A Meta-Analysis of Neuroimaging Studies of Empathy
Source: Front Behav Neurosci. 2018 Nov 27;12:289. doi: 10.3389/fnbeh.2018.00289 (PMC6277791; doi:10.3389/fnbeh.2018.00289)
Supplement: Supplementary file 2 [file Table_2.pdf]

TABLE S2 | Peaks of activation (maximum ALE value) for the brain areas that are consistently activated when contrasting an empathy condition with baseline or a neutral condition. Primary anatomical labels (printed first, starting with a capital letter) are derived from the Talairach Daemon (talairach.org).

Anatomical label		BA	x	y	z	Cluster size[i]
Empathy (all studies together; 1915 foci)						
Frontal Lobe						
L/R	Medial Frontal Gyrus (mid cingulate, supplementary motor area)	32	-4	16	44	10848
L/R	Medial Frontal Gyrus	9	-2	52	28	808
L	Middle Frontal Gyrus	9	-52	18	28	48
R	Inferior Frontal Gyrus	13	50	30	-2	6760
R	Inferior Frontal Gyrus	9	52	8	32	5464
L	Precentral Gyrus	6	-42	2	44	296
Limbic lobe / subcortical regions						
L/R	Cingulate Gyrus (mid / posterior)	24	0	-18	38	432
L/R	Cingulate Gyrus (posterior)	31	-2	-58	32	696
L	Insula (anterior, inferior frontal gyrus)	13	-40	12	-4	15224
R	Claustrum (anterior/mid insula)		42	4	0	832
L	Amygdala		-20	-8	-14	1672
R	Parahippocampal Gyrus / Amygdala		22	-6	-16	2016
L	Caudate		-12	6	12	400
L	Caudate		-12	12	2	296
R	Lentiform Nucleus, Globus Pallidus		16	6	4	1016
L	Thalamus, Medial Dorsal Nucleus		-8	-12	10	136
L	Thalamus, Pulvinar		-16	-26	2	112
L/R	Brainstem (midbrain)		-2	-29	-6	2000
Parietal lobe						
L	Postcentral Gyrus	40	-58	-22	24	4952
L	Inferior Parietal Lobule	40	-38	-38	44	96
L	Inferior Parietal Lobule	40	-38	-48	58	88
L	Inferior Parietal Lobule	40	-60	-42	26	16
R	Inferior Parietal Lobule	40	62	-20	34	3616
R	Precuneus	7	32	-54	58	520
L	Supramarginal Gyrus	40	-52	-50	30	16
Temporal lobe						
L	Fusiform Gyrus	37	-42	-54	-20	600
R	Fusiform Gyrus	37	48	-66	-6	7272
L	Superior Temporal Gyrus	38	-48	10	-36	144
R	Superior Temporal Gyrus	22	54	-22	-10	144
R	Superior Temporal Gyrus	22	60	-42	10	144
R	Superior Temporal Gyrus	38	46	14	-36	560

R	Superior Temporal Gyrus	38	52	8	-26	8
R	Middle Temporal Gyrus		52	-34	-2	488
Occipital lobe						
R	Lingual Gyrus	18	22	-86	-6	416
L	Middle Occipital Gyrus	18	-32	-92	0	480
L	Middle Occipital Gyrus	37	-48	-72	-2	5712
R	Middle Occipital Gyrus	18	34	-84	4	824

Coordinates are in MNI [Montreal Neurologic Institute] space; BA = Brodmann Area; cluster size is in mm^3. [i] Chosen minimum cluster-size was 8 mm^3

TABLE S3 | Peaks of activation for the brain areas that are consistently activated when contrasting an empathy for pain condition with a baseline or neutral condition; an empathy for non-pain negative affective states with a baseline or neutral condition; and conjunction and contrast analyses to identify commonalities and differences in neural circuitry between the two types of empathy

Anatomical label		BA	x	y	z	Cluster size [i]
Empathy for pain (874 foci)						
Frontal lobe						
R	Middle Frontal Gyrus	46	50	22	16	32
R	Middle Frontal Gyrus	6	52	8	42	16
R	Inferior Frontal Gyrus	13	38	10	-20	8
R	Inferior Frontal Gyrus	44	60	12	16	872
R	Inferior Frontal Gyrus	47	34	10	-22	24
Limbic lobe / subcortical regions						
L/R	Cingulate Gyrus (mid cingulate, supplementary motor area)	32	-4	20	38	8712
L	Insula (anterior, inferior frontal gyrus)	13	-40	12	-6	11680
R	Claustrum (mid insula)		42	4	0	4608
L	Parahippocampal Gyrus / Amygdala		-20	-6	-14	192
L	Caudate		-12	12	2	256
R	Lentiform Nucleus, Globus Pallidus		16	6	4	440
R	Lentiform Nucleus, Globus Pallidus		22	-4	-14	280
L	Thalamus, Pulvinar		-4	-30	-2	432
L	Thalamus, Pulvinar		-14	-26	4	8
L/R	Brainstem, Substania Nigra		-6	-28	-18	80
Parietal lobe						
L	Postcentral Gyrus	40	-58	-22	24	5528
L	Inferior Parietal Lobule	40	-38	-48	60	136
L	Inferior Parietal Lobule	40	-38	-38	44	56
R	Inferior Parietal Lobule	40	62	-20	34	4464
R	Precuneus	7	32	-54	58	1136
Temporal / occipital lobe						
R	Fusiform Gyrus	37	48	-64	-8	3192
R	Superior Temporal Gyrus	22	50	-34	0	8
L	Inferior Temporal Gyrus	37	-46	-70	-2	3056
R	Lingual Gyrus	18	18	-90	-2	96
L	Middle Occipital Gyrus	18	-32	-94	0	328
R	Middle Occipital Gyrus	18	34	-86	4	168
Cerebellum						
R	Cerebellum, posterior lobe		36	-62	-24	128
Empathy for non-pain negative affective states (678 foci)						
Frontal lobe						

L	Medial Frontal Gyrus (mid cingulate)	32	-6	14	48	344
R	Medial Frontal Gyrus (mid cingulate)	6	8	10	50	120
L	Medial Frontal Gyrus (anterior)	9	-4	52	22	48
L	Inferior Frontal Gyrus	47	-48	28	-8	1208
L	Inferior Frontal Gyrus	45	-54	24	12	208
R	Inferior Frontal Gyrus		50	30	-4	800
R	Inferior Frontal Gyrus	46	54	34	12	712
L	Superior Frontal Gyrus	6	-6	18	64	64
R	Superior Frontal Gyrus	6	8	12	66	8
L	Precentral Gyrus	6	-42	2	44	120
L	Precentral Gyrus	44	-46	20	4	88
R	Precentral Gyrus	6	52	8	34	336
Limbic lobe / subcortical regions						
L	Claustrum (anterior insula)		-28	24	2	168
L	Parahippocampal Gyrus / Amygdala		-20	-8	-16	592
R	Parahippocampal Gyrus / Amygdala		22	-4	-16	832
L	Caudate		-12	6	12	272
L	Parahippocampal Gyrus / Amygdala	34	-20	0	-22	8
R	Lentiform Nucleus, Globus Pallidus		14	0	0	8
R	Caudate		14	4	12	32
L	Brainstem (midbrain)		-2	-30	-6	600
Parietal lobe						
L	Inferior Parietal Lobule	40	-60	-42	26	8
Temporal / occipital lobe						
R	Inferior Temporal Gyrus	19	50	-56	2	16
R	Middle Temporal Gyrus	37	52	-54	4	8
L	Middle Temporal Gyrus	39	-52	-60	12	8
R	Superior Temporal Gyrus	38	46	14	-36	320
L	Superior Temporal Gyrus	38	-48	10	-34	216
R	Fusiform Gyrus	37	44	-48	-20	200
L	Fusiform Gyrus	37	-42	-44	-18	104
R	Middle Occipital Gyrus	19	30	-92	10	48
Cerebellum						
L	Cerebellum, Anterior Lobe		-34	-54	-24	32
Empathy for pain ∩ Empathy for non-pain negative affective states						
Frontal lobe						
L	Medial Frontal Gyrus (mid cingulate)	32	-4	16	48	160
R	Inferior Frontal Gyrus	45	52	32	-2	464
R	Inferior Frontal Gyrus	9	54	8	30	32
R	Superior Frontal Gyrus	6	4	10	54	8
R	Superior Frontal Gyrus	6	8	12	66	8

L	Precentral Gyrus	44	-46	20	4	80
Limbic lobe / subcortical regions						
L	Insula (anterior)	13	-36	22	-4	328
L	Claustrum (anterior insula)		-28	24	4	80
L	Thalamus, Pulvinar		-4	-30	-4	256
R	Lentiform Nucleus, Globus Pallidus		22	-4	-14	248
L	Parahippocampal Gyrus / Amygdala		-20	-6	-14	160
Empathy for pain > Empathy for non-pain negative affective states						
Frontal lobe						
L	Medial Frontal Gyrus (supplementary motor area)	6	0	2	68	72
L	Precentral Gyrus	6	-56	4	34	256
Limbic lobe / subcortical regions						
L/R	Cingulate Gyrus (mid cingulate)	24	-2	21	36	4264
L	Insula (anterior, mid, inferior frontal gyrus)	13	-50	5	10	5496
R	Claustrum (anterior, mid insula)		43	4	-3	2256
Parietal lobe						
L	Postcentral Gyrus	40	-58	-25	34	4328
R	Postcentral Gyrus	40	62	-21	29	3320
R	Precuneus	7	33	-48	57	992
Temporal / occipital lobe						
R	Fusiform Gyrus	37	53	-61	-9	1176
L	Middle Occipital Gyrus	37	-46	-64	-5	1232
Empathy for non-pain negative affective states > Empathy for pain						
No significant clusters						

Coordinates are in MNI [Montreal Neurologic Institute] space; BA = Brodmann Area; Cluster size is in mm^3. [i] Chosen minimum cluster-size was 8 mm^3

TABLE S4 | Peaks of activation for the brain areas that are consistently activated when contrasting an empathy for pain condition using facial pain expressions with a baseline or neutral condition; an empathy for pain condition using acute pain inflictions to body parts with a baseline or neutral condition; and conjunction and contrast analyses to identify commonalities and differences in neural circuitry between these two types of paradigms to elicit empathy for pain

Anatomical label		BA	x	y	z	Cluster size [i]
Empathy for pain using acute pain inflictions (605 foci)						
Frontal lobe						
L/R	Medial Frontal Gyrus (supplementary motor area)	6	0	4	64	1976
L	Inferior Frontal Gyrus	44	-52	10	18	2584
L	Inferior Frontal Gyrus	46	-42	42	8	104
R	Inferior Frontal Gyrus	13	38	10	-20	112
R	Inferior Frontal Gyrus	44	60	12	14	352
R	Inferior Frontal Gyrus	45	52	32	0	8
R	Middle Frontal Gyrus	6	52	8	42	88
Limbic lobe / subcortical regions						
L/R	Cingulate Gyrus (mid-cingulate)	32	-4	22	36	3768
L	Insula (anterior, inferior frontal gyrus)	13	-40	12	-4	4920
R	Insula (anterior, inferior frontal gyrus)	13	38	24	0	3040
L	Insula (mid / posterior)	13	-40	-2	14	64
R	Parahippocampal Gyrus / Amygdala		22	-6	-14	64
R	Caudate		14	6	4	112
R	Brainstem, Red Nucleus		4	-28	-8	40
Parietal lobe						
L	Inferior Parietal Lobule	40	-56	-26	36	5432
L	Inferior Parietal Lobule	40	-38	-48	60	280
R	Inferior Parietal Lobule	40	62	-20	34	4584
R	Precuneus	7	32	-54	58	1344
Temporal / Occipital lobe						
R	Fusiform Gyrus	37	50	-64	-10	1112
L	Inferior Temporal Gyrus	37	-46	-68	-2	1752
L	Middle Occipital Gyrus	18	-32	-94	0	112
R	Middle Occipital Gyrus	18	34	-86	2	288
Cerebellum						
R	Cerebellum, Posterior Lobe		36	-64	-24	112
Empathy for pain using facial pain expressions (260 foci)						
Limbic lobe / subcortical regions						
L	Cingulate Gyrus (mid-cingulate)	32	-4	18	42	232
L	Insula (anterior)	13	-40	12	-6	40
Temporal / Occipital lobe						
L	Inferior Temporal Gyrus	37	-46	-74	0	168

R	Inferior Temporal Gyrus	37	48	-70	0	280
Facial pain expressions ∩ Acute pain inflictions						
Limbic lobe / subcortical regions						
L	Cingulate Gyrus (mid-cingulate)	32	-4	18	42	160
L	Insula (anterior)	13	-40	12	-6	40
Temporal / Occipital lobe						
L	Inferior Temporal Gyrus	37	-46	-72	0	64
R	Fusiform Gyrus (inferior temporal gyrus)	37	48	-64	-6	56
Acute pain inflictions > Facial pain expressions						
Frontal lobe						
L/R	Medial Frontal Gyrus	8	2	38	40	192
Parietal lobe						
R	Superior Parietal Lobule	7	36	-56	62	176
R	Superior Parietal Lobule	7	35	-52	64	16
L	Inferior Parietal Lobule	40	-51	-29	37	1576
R	Inferior Parietal Lobule	40	57	-24	39	2208
Painful facial expressions > Acute pain inflictions						
No significant clusters						

Coordinates are in MNI [Montreal Neurologic Institute] space; BA = Brodmann Area; Cluster size is in mm^3. [i] Chosen minimum cluster-size was 8 mm^3

TABLE S5 | Peaks of activation for the brain areas that are consistently activated when contrasting a cognitive/evaluative empathy paradigm with a baseline or neutral condition; a perceptual/affective empathy paradigm with a baseline or neutral condition; and conjunction and contrast analyses to identify commonalities and differences in neural circuitry between these two types of paradigms to elicit empathy

Anatomical label		BA	x	y	z	Cluster size
Cognitive/evaluative empathy paradigms (1129 foci)						
Frontal Lobe						
L	Medial Frontal Gyrus	6	-4	54	30	392
R	Middle Frontal Gyrus	46	54	36	14	40
L	Inferior Frontal Gyrus	44	-54	12	12	1408
L	Inferior Frontal Gyrus	45	-54	24	12	248
L	Inferior Frontal Gyrus	47	-48	30	-8	288
R	Inferior Frontal Gyrus	44	58	12	18	704
R	Inferior Frontal Gyrus	45	52	30	-4	1536
R	Inferior Frontal Gyrus	47	36	30	-4	48
R	Inferior Frontal Gyrus	6	50	6	32	136
Limbic lobe / subcortical regions						
L	Cingulate Gyrus (anterior, mid)	32	-2	26	24	8
L	Cingulate Gyrus (mid, L/R supplementary motor area)	32	-4	20	40	5824
L/R	Cingulate Gyrus (mid, posterior)	24	0	-16	38	488
L/R	Cingulate Gyrus (posterior, precuneus)	31	-2	-58	32	552
L	Parahippocampal Gyrus / Amygdala		-20	-8	-16	488
R	Parahippocampal Gyrus / Amygdala		22	-4	-16	208
L	Insula (anterior)	13	-42	12	-6	3704
L	Insula (mid)	13	-40	0	6	32
R	Claustrum (mid / posterior insula)		40	-6	-8	24
R	Lentiform Nucleus, Globus Pallidus		16	6	4	1080
L	Caudate		-12	12	2	648
L	Thalamus		-20	-26	-2	8
L	Thalamus		-18	-24	0	8
L	Thalamus, Pulvinar (brainstem)		-4	-30	-2	760
Parietal lobe						
L	Postcentral Gyrus	40	-58	-22	24	2720
R	Inferior Parietal Lobule	40	62	-22	34	1904
R	Precuneus	7	32	-54	58	296
L	Supramarginal Gyrus	40	-52	-50	30	40
Temporal / occipital lobe						
R	Fusiform Gyrus	37	42	-54	-14	16
L	Superior Temporal Gyrus	38	-48	10	-36	312
L	Superior Temporal Gyrus	39	-54	-62	22	80

R	Superior Temporal Gyrus	22	52	-32	-2	16
R	Superior Temporal Gyrus	38	46	14	-36	720
L	Middle Temporal Gyrus	21	-60	-10	-14	8
L	Middle Temporal Gyrus	39	-48	-66	26	8
R	Middle Temporal Gyrus	37	54	-62	0	568
L	Inferior Temporal Gyrus	37	-48	-70	-2	1056
R	Lingual Gyrus	18	22	-86	-6	88
L	Middle Occipital Gyrus	18	-32	-92	2	32
R	Middle Occipital Gyrus	19	30	-92	10	256
Cerebellum						
L	Cerebellum, Anterior Lobe		-36	-54	-24	40
R	Cerebellum, Anterior Lobe		34	-62	-26	152
Perceptual/affective empathy paradigms (760 foci)						
Frontal Lobe						
L	Medial Frontal Gyrus (supplementary motor area)	6	-2	8	56	40
R	Medial Frontal Gyrus (mid-cingulate / supplementary motor area)	6	8	12	52	184
L	Medial Frontal Gyrus	9	2	50	28	8
R	Middle Frontal Gyrus	46	48	22	18	32
R	Middle Frontal Gyrus	6	50	6	40	680
R	Middle Frontal Gyrus	9	56	20	26	16
L	Inferior Frontal Gyrus	44	-50	10	18	216
L	Inferior Frontal Gyrus	47	-38	24	-14	2032
L	Inferior Frontal Gyrus	9	-52	18	26	56
R	Inferior Frontal Gyrus	44	62	12	14	200
L	Precentral Gyrus	6	-58	8	30	224
Limbic lobe / subcortical regions						
L	Insula (anterior)	13	-32	26	4	136
R	Insula (anterior)	13	42	24	0	2424
R	Claustrum (mid cingulate)		42	2	-2	88
L	Amygdala		-20	-8	-14	616
R	Parahippocampal Gyrus / Amygdala		22	-6	-14	1264
L/R	Brainstem, Red Nucleus		-4	-28	-8	824
Parietal lobe						
R	Postcentral Gyrus	2	62	-20	36	856
L	Inferior Parietal Lobule	40	-56	-24	36	1456
Temporal / occipital lobe						
L	Fusiform Gyrus	19	-40	-80	-10	16
R	Fusiform Gyrus	37	44	-48	-20	344
R	Fusiform Gyrus	37	48	-66	-8	3064
L	Inferior Temporal Gyrus	37	-46	-70	-2	2104

L	Middle Temporal Gyrus	21	-52	-50	6	88
R	Superior Temporal Gyrus	21	56	-22	-8	96
Cognitive/evaluative ∩ Perceptual/affective empathy paradigms						
Frontal Lobe						
L	Medial Frontal Gyrus (supplementary motor area)	6	-2	8	56	16
R	Medial Frontal Gyrus (supplementary motor area)	6	6	10	54	8
L	Inferior Frontal Gyrus	44	-50	10	16	56
L	Inferior Frontal Gyrus	47	-38	22	-10	8
R	Inferior Frontal Gyrus	13	48	30	-2	672
R	Inferior Frontal Gyrus	44	60	12	14	32
L	Precentral Gyrus	44	-46	18	2	960
R	Precentral Gyrus	6	52	8	34	48
Limbic lobe / subcortical regions						
L	Insula (anterior)	13	-32	26	4	96
L	Insula (anterior)	13	-38	24	-2	24
L	Amygdala		-20	-8	-14	424
R	Parahippocampal Gyrus / Amygdala		22	-4	-16	208
L/R	Brainstem (midbrain)		-2	30	-6	248
Parietal lobe						
R	Postcentral Gyrus	2	62	-20	36	808
L	Inferior Parietal Lobule	40	-56	-24	36	1296
Temporal / occipital lobe						
R	Inferior Temporal Gyrus	37	50	-66	-4	408
L	Middle Occipital Gyrus	37	-48	-72	-2	848
Cognitive/evaluative > Perceptual/affective empathy paradigms						
No significant clusters						
Perceptual/affective > Cognitive/evaluative empathy paradigms						
Frontal lobe						
R	Inferior Frontal Gyrus (anterior insula)	13	43	26	4	32

Coordinates are in MNI [Montreal Neurologic Institute] space; BA = Brodmann Area; Cluster size is in mm^3. [i] Chosen minimum cluster-size was 8 mm^3

TABLE S6 | Peaks of activation for the brain areas that are consistently activated when contrasting a pain-specific cognitive/evaluative empathy paradigm with baseline or neutral condition; a perceptual/affective empathy for pain paradigm with baseline or neutral condition; and conjunction and contrast analyses to identify commonalities and differences in neural circuitry between these two types of paradigms to elicit empathy for pain

Anatomical label		BA	x	y	z	Cluster size
Cognitive/evaluative paradigms for empathy for pain (508 foci)						
Frontal Lobe						
L/R	Medial Frontal Gyrus (supplementary motor area)	6	2	6	62	1584
L	Middle Frontal Gyrus	10	-42	44	8	120
R	Inferior Frontal Gyrus	44	58	12	16	528
R	Inferior Frontal Gyrus	45	52	32	0	248
R	Inferior Frontal Gyrus	47	36	30	-4	16
Limbic lobe / subcortical regions						
L	Cingulate Gyrus (mid)	32	-4	20	38	4176
L	Insula (anterior, inferior frontal gyrus)	13	-42	12	-6	5888
R	Insula (anterior, mid)		44	6	-2	368
R	Claustrum		42	-6	-8	192
R	Lentiform Nucleus, Globus Pallidus		16	6	4	584
L	Caudate		-14	14	2	288
L/R	Thalamus, Pulvinar (brainstem, midbrain)		-4	-30	0	152
Parietal lobe						
L	Postcentral Gyrus	40	-58	-22	24	3800
R	Inferior Parietal Lobule	40	62	-22	34	2368
R	Precuneus	7	32	-54	58	688
Temporal / occipital lobe						
R	Fusiform Gyrus	37	52	-62	-10	152
L	Inferior Temporal Gyrus	37	-46	-68	0	264
L	Middle Occipital Gyrus	18	-32	-94	0	64
Cerebellum						
R	Cerebellum, Posterior Lobe		36	-64	-24	64
Perceptual/affective paradigms for empathy for pain (344 foci)						
Frontal Lobe						
L	Medial Frontal Gyrus	8	-4	34	38	16
L	Inferior Frontal Gyrus	44	-50	10	18	272
L	Precentral Gyrus	6	-58	8	30	272
Limbic lobe / subcortical regions						
L	Insula (anterior, inferior frontal gyrus)	13	-32	26	4	16
R	Insula (anterior, inferior frontal gyrus)	13	40	24	0	616
L	Claustrum (anterior, mid insula)		-38	12	-2	216

R	Claustrum (mid insula)		42	2	-2	232
R	Parahippocamal Gyrus / Amygdala		22	-6	-14	448
L	Parahippocamal Gyrus / Amygdala		-22	-6	-14	72
L/R	Brainstem (midbrain)		2	-30	-8	208
Parietal lobe						
L	Inferior Parietal Lobule	40	-56	-24	36	1280
R	Inferior Parietal Lobule	40	62	-20	34	976
Temporal / occipital lobe						
R	Fusiform Gyrus	37	48	-64	-8	1560
L	Inferior Temporal Gyrus	37	-44	-70	-4	1384
Cerebellum						
R	Cerebellum, Posterior Lobe		16	-76	-50	8
Cognitive/evaluative ∩ Perceptual/affective paradigms for empathy for pain						
Frontal Lobe						
L	Inferior Frontal Gyrus	44	-50	10	16	152
Limbic lobe / subcortical regions						
L	Claustrum		-38	12	-2	208
R	Claustrum		42	4	0	24
Parietal lobe						
L	Inferior Parietal Lobule	40	-56	-24	36	1232
R	Inferior Parietal Lobule	40	62	-20	34	960
Temporal / occipital lobe						
R	Fusiform Gyrus	37	50	-64	-10	88
L	Inferior Temporal Gyrus	37	-46	-68	0	240
Cognitive/evaluative > Perceptual/affective						
L	Cingulate Gyrus (mid)	32	-7	20	41	216
Perceptual/affective > Cognitive/evaluative						
R	Inferior Frontal Gyrus (anterior insula)	13	43	26	4	32

Coordinates are in MNI [Montreal Neurologic Institute] space; BA = Brodmann Area; Cluster size is in mm^3. [i] Chosen minimum cluster-size was 8 mm^3
