# Supplementary material for: Is Empathy for Pain Unique in Its Neural Correlates? A Meta-Analysis of Neuroimaging Studies of Empathy
Source: Front Behav Neurosci. 2018 Nov 27;12:289. doi: 10.3389/fnbeh.2018.00289 (PMC6277791; doi:10.3389/fnbeh.2018.00289)
Supplement: Supplementary file 3 [file Data_Sheet_1.pdf]

SUPPLEMENTARY METHODS

Full search terms per library are listed below:

PubMed (https://www.ncbi.nlm.nih.gov/pubmed?otool=stanford&holding=F1000,F1000M)

("empathy" [mesh] OR empath* [tw]) AND (fmri [tw] OR imaging [ti] OR image [ti] OR images [ti] OR asl [tw] OR "Diagnostic Imaging" [mesh] OR pet [tw] OR "Positron Emission Tomography" [tw] OR "pet scan" [tw] OR "brain mapping" [mesh] OR "brain mapping" [tw] OR "neuroimaging" [mesh] OR neuroimaging [tw] OR "brain imaging" [tw] OR "arterial spin labeling" [tw] OR "Magnetic Resonance Imaging" [mesh] OR "Magnetic Resonance Imaging" [tw] OR mri [tw] OR "Functional Neuroimaging" [mesh]) AND english [lang] NOT ("animals" [mesh] NOT "humans" [mesh])

Embase (https://www.embase.com/#search)

('empathy'/exp OR empath*:ti,ab,kw) AND (fmri:ti,ab,kw OR imaging:ti OR image:ti OR images:ti OR asl:ti,ab,kw OR ‘diagnostic imaging'/exp OR pet:ti,ab,kw OR 'positron emission tomography'/exp OR ‘pet scan’:ti,ab,kw OR 'brain mapping'/exp OR "brain mapping":ti,ab,kw OR 'neuroimaging'/exp OR neuroimaging:ti,ab,kw OR "brain imaging”:ti,ab,kw OR "arterial spin labeling”:ti,ab,kw OR 'arterial spin labeling'/exp OR 'nuclear magnetic resonance imaging'/exp OR "Magnetic Resonance Imaging":ti,ab,kw OR mri:ti,ab,kw) AND [english]/lim

Cochrane Library (http://cochranelibrary-wiley.com/cochranelibrary/search/)

Empath* AND (fmri OR imaging OR image OR images OR asl OR ‘diagnostic imaging' OR pet OR 'positron emission tomography' OR ‘pet scan’ OR 'brain mapping' OR "brain mapping" OR 'neuroimaging' OR neuroimaging OR "brain imaging” OR "arterial spin labeling” OR 'arterial spin labeling' OR 'nuclear magnetic resonance imaging' OR "Magnetic Resonance Imaging" OR mri)

PsychInfo (http://ovidsp.tx.ovid.com/sp-3.27.1a/ovidweb.cgi)

1. exp Empathy/ OR empath*.ti,tm,id,sh.
2. fmri.ti,tm,id,sh OR imaging.ti OR image.ti OR images.ti OR asl.ti,tm,id,sh OR pet.ti,tm,id,sh OR exp Positron Emission Tomography/ OR "pet scan".ti,tm,id,sh. OR exp stereotaxic atlas/ OR "brain mapping".ti,tm,id,sh. OR exp NEUROIMAGING/ OR neuroimaging.ti,tm,id,sh. OR "brain imaging".ti,tm,id,sh. OR "arterial spin labeling".ti,tm,id,sh. OR exp Magnetic Resonance Imaging/ OR "magnetic resonance".ti,tm,id,sh OR mri.ti,tm,id,sh.
3. 1. and 2.
4. Limit 3 to English language
